# Supplementary material for: Assessing the impact of a combined nutrition counselling and cash transfer intervention on women’s empowerment in rural Bangladesh: a randomised control trial protocol
Source: BMJ Open. 2021 Jun 9;11(6):e044263. doi: 10.1136/bmjopen-2020-044263 (PMC8191606; doi:10.1136/bmjopen-2020-044263)
Supplement: Supplementary data [file bmjopen-2020-044263supp001.pdf]

Appendix

Table 1: SCC women's empowerment summary index components

**Shonjibon Trial resources**	**Pathway**	**Outcome variable / index components**
mHealth app, access to call centre (BCC on Nutrition, IYCF and livelihoods) and mobile phone<br><br>Unconditional cash transfer (delivered on Bkash mobile banking app)	Instrumental Agency (Power to)	1. Control over use of income (G2)<br>2. Input in production decisions (G2)
	Intrinsic agency (power within)	3. Autonomy in income (G8(a))<br>4. Decision-making power on nutrition and health care (X)<br>5. Respect among household members (G7)<br>6. Attitudes toward IPV (G9)

Table 2: Data collection timeline

**Methods**	**Participants**	**timeline**
Semi-structured in-depth interview at the household level		
In-depth interviews with women	1) Pregnant women<br>2) Mothers of children <1 month<br>3) Mothers 1-5 months<br>4) Mothers of children 6 – 59 months	➢ At the start of SCC Trial<br>➢ Endline
In-depth interviews with family members	1) Mother or mother-in-law of women<br>2) Husband of women	➢ At the start of SCC Trial<br>➢ Endline

Kirkwood EK, *et al. BMJ Open* 2021; 11:e044263. doi: 10.1136/bmjopen-2020-044263

	3) Other key family members as directed by the woman interviewed	
Pro-WEAI Survey questions		
Survey questionnaires	Female intervention recipients	➢ Baseline<br>➢ Endline

Table 3: Social Desirability Bias

**Directions: Read each item and decide whether it is true (T) or false (F) for you.**			
		**True**	**False**
1	It is sometimes hard for me to go on with my work if I am not encouraged.		
2	I sometimes feel resentful when I don't get my way		
3	On a few occasions, I have given up doing something because I thought too little of my ability		
4	There have been times when I felt like rebelling against people in authority even though I knew they were right		
5	No matter who I'm talking to, I'm always a good listener		
6	There have been occasions when I took advantage of someone		
7	I'm always willing to admit it when I make a mistake		
8	I sometimes try to get even rather than forgive and forget		
9	I am always courteous, even to people who are disagreeable		
10	I have never been irked when people expressed ideas very different from my own		
11	There have been times when I was quite jealous of the good fortune of others		
12	I am sometimes irritated by people who ask favours of me		
13	I have never deliberately said something that hurt someone's feelings		

Kirkwood EK, *et al. BMJ Open* 2021; 11:e044263. doi: 10.1136/bmjopen-2020-044263
