# Supplementary material for: Assessing the impact of a combined nutrition counselling and cash transfer intervention on women’s empowerment in rural Bangladesh: a randomised control trial protocol
Source: BMJ Open. 2021 Jun 9;11(6):e044263. doi: 10.1136/bmjopen-2020-044263 (PMC8191606; doi:10.1136/bmjopen-2020-044263)
Supplement: Author's manuscript [file bmjopen-2020-044263.draft_revisions.pdf]

BMJ Open is committed to open peer review. As part of this commitment we make the peer review history of every article we publish publicly available.

When an article is published we post the peer reviewers' comments and the authors' responses online. We also post the versions of the paper that were used during peer review. These are the versions that the peer review comments apply to.

The versions of the paper that follow are the versions that were submitted during the peer review process. They are not the versions of record or the final published versions. They should not be cited or distributed as the published version of this manuscript.

BMJ Open is an open access journal and the full, final, typeset and author-corrected version of record of the manuscript is available on our site with no access controls, subscription charges or pay-per-view fees (http://bmjopen.bmj.com).

If you have any questions on BMJ Open's open peer review process please email info.bmjopen@bmj.com

BMJ Open

Assessing the impact of a combined nutrition counselling and cash transfer intervention on women's empowerment in rural Bangladesh: A randomised control trial protocol.

Journal:	*BMJ Open*
Manuscript ID	bmjopen-2020-044263
Article Type:	Protocol
Date Submitted by the Author:	28-Aug-2020
Complete List of Authors:	Kirkwood, Elizabeth; The University of Sydney Faculty of Medicine and Health, Sydney School of Public Health;<br>Dibley, Michael; The University of Sydney, School of Public Health<br>Hoddinott, John Frederick ; Cornell University,<br>Huda, Tanvir ; The University of Sydney, Sydney School of Public Health<br>Laba, Tracey; University of Technology Sydney, Centre for Health Economics Research and Evaluation<br>Tahsina, Tazeen; International Centre for Diarrhoeal Disease Research Bangladesh, Maternal and Child Health Division<br>Hasan, Mohammad; International Centre for Diarrhoeal Disease Research Bangladesh<br>Iqbal, Afrin; International Centre for Diarrhoeal Disease Research Bangladesh<br>Khan, Jasmin; International Centre for Diarrhoeal Disease Research Bangladesh<br>Ali, Nazia; International Centre for Diarrhoeal Disease Research Bangladesh<br>Ullah, Saad; International Centre for Diarrhoeal Disease Research Bangladesh<br>Goodwin, Nicholas; The University of Sydney<br>Muthayya, Sumithra; The Sax Institute<br>Islam, M; International Centre for Diarrhoeal Disease Research Bangladesh, Nutrition and Clinical Services Division<br>Ara, Gulshan; International Centre for Diarrhoeal Disease Research Bangladesh, NCSD<br>Agho, Kingsley ; Western Sydney University, School of Science and Health,<br>Arifeen, Shams E.; ICDDR B, Maternal and Child Health Division (MCHD)<br>Alam, Ashraful; University of Sydney, School of Public Health
Keywords:	PUBLIC HEALTH, Protocols & guidelines < HEALTH SERVICES ADMINISTRATION & MANAGEMENT, Clinical trials < THERAPEUTICS

SCHOLARONE™
Manuscripts

I, the Submitting Author has the right to grant and does grant on behalf of all authors of the Work (as defined in the below author licence), an exclusive licence and/or a non-exclusive licence for contributions from authors who are: i) UK Crown employees; ii) where BMJ has agreed a CC-BY licence shall apply, and/or iii) in accordance with the terms applicable for US Federal Government officers or employees acting as part of their official duties; on a worldwide, perpetual, irrevocable, royalty-free basis to BMJ Publishing Group Ltd ("BMJ") its licensees and where the relevant Journal is co-owned by BMJ to the co-owners of the Journal, to publish the Work in this journal and any other BMJ products and to exploit all rights, as set out in our licence.

The Submitting Author accepts and understands that any supply made under these terms is made by BMJ to the Submitting Author unless you are acting as an employee on behalf of your employer or a postgraduate student of an affiliated institution which is paying any applicable article publishing charge ("APC") for Open Access articles. Where the Submitting Author wishes to make the Work available on an Open Access basis (and intends to pay the relevant APC), the terms of reuse of such Open Access shall be governed by a Creative Commons licence – details of these licences and which Creative Commons licence will apply to this Work are set out in our licence referred to above.

Other than as permitted in any relevant BMJ Author's Self Archiving Policies, I confirm this Work has not been accepted for publication elsewhere, is not being considered for publication elsewhere and does not duplicate material already published. I confirm all authors consent to publication of this Work and authorise the granting of this licence.

For peer review only

Title

Assessing the impact of a combined nutrition counselling and cash transfer intervention on women's empowerment in rural Bangladesh: A randomised control trial protocol.

Authors

Elizabeth K. Kirkwood - ORCID 0000-0001-8603-4903

The University of Sydney, Sydney School of Public Health; Edward Ford Building, NSW, 2006, Australia.

elizabeth.kirkwood@sydney.edu.au

Michael J. Dibley - ORCID 0000-0002-1554-5180

The University of Sydney, Sydney School of Public Health; Edward Ford Building, NSW, 2006, Australia michael.dibley@sydney.edu.au

John Hoddinott – ORCID 0000-0002-0590-3917

Cornell University

Tanvir Huda – ORCID 0000-0002-8996-4361

The University of Sydney, Sydney School of Public Health; Edward Ford Building, NSW, 2006, Australia.

Tracey-Lea Laba – ORCID 0000-0002-5182-9092

University of Technology Sydney

Tazeen Tahsina – ORCID 0000-0001-5899-8422

International Centre for Diarrhoeal Disease Research, Bangladesh

Mohammad Mehedi Hasan – ORCID 0000-0003-0630-290X

International Centre for Diarrhoeal Disease Research, Bangladesh

Afrin Iqbal – ORCID 0000-0002-1859-8405

International Centre for Diarrhoeal Disease Research, Bangladesh

Jasmin Khan – ORCID 0000-0003-2022-6448

International Centre for Diarrhoeal Disease Research, Bangladesh

Nazia Binte Ali – ORCID 0000-0001-5010-9563

For peer review only - http://bmjopen.bmj.com/site/about/guidelines.xhtml

For peer review only

International Centre for Diarrhoeal Disease Research, Bangladesh

Saad Ullah - ORCID 0000-0001-7450-6483

International Centre for Diarrhoeal Disease Research, Bangladesh

Nicholas Goodwin – ORCID 0000-0003-2476-6310

The University of Sydney, Sydney School of Public Health; Edward Ford Building, NSW, 2006, Australia.

Sumithra Muthayya– ORCID 0000-0003-1645-9206

Sax Institute

Munirul Islam – ORCID 0000-0002-8780-8760

International Centre for Diarrhoeal Disease Research, Bangladesh

Gulshan Ara– ORCID 0000-0002-3745-5952

International Centre for Diarrhoeal Disease Research, Bangladesh

Kingsley Emwinyore Agho – ORCID 0000-0003-4111-3207

Western Sydney University

Shams El Arifeen – ORCID 0000-0002-5372-5932

International Centre for Diarrhoeal Disease Research, Bangladesh

Ashraful Alam - ORCID: 0000-0001-7034-1095

The University of Sydney, Sydney School of Public Health; Edward Ford Building, NSW, 2006, Australia neeloy.alam@sydney.edu.au

Corresponding author

Elizabeth K. Kirkwood

The University of Sydney, Sydney School of Public Health; Room 328 Edward Ford Building, NSW, 2006, Australia. Phone: +61 410 517 874

Email: elizabeth.kirkwood@sydney.edu.au

For peer review only - http://bmjopen.bmj.com/site/about/guidelines.xhtml

Abstract

Introduction: Evidence suggests that women's empowerment is an underlying determinant of nutrition outcomes. The level of empowerment experienced by women directly impacts agricultural productivity, household food security and child nutrition. Globally, evidence has shown that interventions targeting nutrition, health and economic development affect women's empowerment.

Objective: This study evaluates the impact of the Shonjibon Cash and Counselling trial that combines nutrition counselling and unconditional cash transfer intervention, delivered on a mobile platform, on women's empowerment in rural Bangladesh.

Methods and analysis: We will use a mixed-methods approach, combining statistical analysis of quantitative data from 2,080 women in a cluster randomised controlled trial examining the impact of nutrition behaviour change communications and cash transfers on child undernutrition. Pregnant participants will be given a smartphone with a customised app, delivering nutrition behaviour change communication messages, receive nutrition counselling via a call centre and an unconditional cash transfer. This study is a component of the Shonjibon Cash and Counselling trial and will measure women's empowerment using a composite indicator based on the Project-Level Women's Empowerment in Agriculture Index, with quantitative data collection at baseline and endline. Thematic analysis of qualitative data, collected through longitudinal interviews with women, husbands, and mothers-in-law, will elicit a local understanding of women's empowerment and the linkages between the intervention and women's empowerment outcomes. This paper describes the study protocol to evaluate women's empowerment in a nutrition-specific and sensitive-intervention using internationally validated, innovative tools and will help fill the evidence gap on pathways of impact, highlighting areas to target for future programming.

Ethics and dissemination:

For peer review only - http://bmjopen.bmj.com/site/about/guidelines.xhtml

Ethical approval has been obtained from the International Centre for Diarrhoeal Disease Research (Ref. PR 17106) and The University of Sydney (Ref: 2019/840). Findings from this study will be shared in Bangladesh with dissemination sessions in-country, internationally at conferences, and published in peer-reviewed journals.

Word count: 4703

Keywords

Women's empowerment, nutrition, cash transfer, counselling, BCC, mHealth, protocol, Bangladesh.

Strengths and limitations of this study

- This paper describes the study protocol evaluating women's empowerment in a nutrition-specific and -sensitive RCT and will help fill the evidence gap on pathways of impact for women's empowerment and highlight areas to target for future policy and programming.
- Formative research guided this mixed-methods approach.
- Designed specifically tailored tools, based on a theory of change and using an internationally validated index, piloted in Bangladesh, indices that can provide global comparison.
- We are not using the Pro-WEAI in full as we interview women only for the quantitative component and will not calculate the Gender Parity Index, which index sheds light on the sense of intrahousehold inequality, however, we will explore this with qualitative research.

Background

To define women's empowerment is not straightforward, nor is it easy to measure across the different domains and phases of a woman's life. Empowerment is "the process of enhancing an individual's or group's capacity to make purposive choices and to transform those choices into desired actions and outcomes"(1). The United Nations defines women's empowerment as having five key components: "women's sense of self-worth; their right to have and to determine choices; their right to have access to opportunities and resources; their right to have the power to control their own lives, both within and outside the home; and their ability to influence the direction of social change to create a more just social and economic order, nationally and internationally" (2).

For peer review only - http://bmjopen.bmj.com/site/about/guidelines.xhtml

Gender, socially constructed roles assigned by sex, is one of the keynote determinants of health outcomes (3), making gender equality and the rights of women an imperative goal in global public health. Gender inequality and restrictive gender norms are, according to the Lancet 2019 series on Gender Equality, Norms and Health "powerful but separate determinants of health and well-being" (3). Sustainable Development Goal (SDG) 5 aims to achieve gender equality and empower all women and girls, ending discrimination in all forms; from violence to forced marriage, full active participation and equal rights in all spheres of life – politically, economically, and socially (4). Out of the 17 SDGs, 11 entail indicators related to gender.

Much progress has been made toward gender equality and the empowerment of women globally. Yet, the potential for women to fully participate as "agents of change" is still limited due to persistent social, economic and political inequalities (5). Women continue to be constrained by norms, beliefs, and customs through which societies differentiate between women and men (6). Contextual forms of oppression include patriarchal societies and institutions, as well as lack of opportunity due to socioeconomic circumstances such as poverty. Violence, including intimate partner violence (IPV), disproportionally affects women. Worldwide, a third of women report experience physical or sexual IPV (7). Women subjected to IPV suffer poor mental and physical health, and their children show poorer health, nutrition and development outcomes (8).

The 2013 Lancet Series on Maternal and Child Nutrition proposed utilising interventions that raise women's overall level of empowerment (9). The series recommends enhancing nutrition sensitivity of programmes by engaging women. Research has shown evidence of the impact of various development interventions on women's empowerment (10-12). Women play a key role in the distribution and access to food at a household level with direct consequences for maternal and child nutritional status (13, 14). Inequitable power relations, based on gender, at the household level, makes women more susceptible to food insecurity (15, 16). A 2018 analysis of surveys from 54 countries observed that in critical aspects of

For peer review only - http://bmjopen.bmj.com/site/about/guidelines.xhtml

family relationships, four out of five women did not hold agency (17). Cunningham et al. (2015) (18) note that women lack not only the resources to make significant decisions leading to better health and nutritional outcomes, but when disempowered, they also lack the autonomy and decision-making power within the household to make these critical decisions, having a multiplier effect on family and community.

Interventions to improve the health and nutrition literacy of women and, in particular, counselling through mobile phone applications can make a positive impact on women's empowerment. Several studies have explored how mobile phone programs influence and enhance communication between women and men. Mobile health (mHealth) interventions have the potential to improve a woman's confidence and decision making skills in communicating with health care professionals. However, a systematic review revealed the need for a further rigorous investigation into mHealth, in terms of implementation and evaluation, to establish whether mHealth programs transform rather than reinforce gender inequalities (19). When a woman is the target of a program and recipient of the mobile phone, this can challenge gender norms within relationships and exacerbate gender disparities (19). There is limited evidence on the adverse effects of mHealth, such as expanding the digital divide and gender-based power imbalances, and this lack of evidence emphasises the need to monitor the impact of mHealth interventions on gender relations and women's empowerment (19).

The Lancet Series on Maternal and Child nutrition also advocates the use of cash transfer and social safety net programmes, which are increasingly the preferred approach to support households living in chronic and extreme poverty (9). When targeted at women, social protection can promote women's economic empowerment and enhance decision-making ability, with the overarching assumption that control over cash will lead to greater investment in children's health and education (9, 20). When women are engaged in nutrition-sensitive programmes that utilise social safety nets, certain aspects of women's empowerment are augmented, such as changes in gender roles and intrahousehold bargaining power (9).

For peer review only - http://bmjopen.bmj.com/site/about/guidelines.xhtml

Developmental interventions and women's empowerment in Bangladesh

Although Bangladesh has made remarkable reductions in rates of poverty, almost 1 in 4 of the population still live in poverty (24.3% in 2016) (21). In 2018, the agriculture sector accounted for 40% of total employment (22). In most developing countries, women account for almost half of the agricultural labour force; however, in Bangladesh, women exceed fifty per cent of the agricultural labour force (23). In rural Bangladesh, social constructs such as gender roles of women and men powerfully drive household food consumption (14). A nationally representative sample from Bangladesh found that women's empowerment increases dietary diversity and availability of calories at a household level (24, 25). The Government of Bangladesh and development organisations have shown a keen interest in combining nutrition counselling and cash transfers to improve maternal and child nutrition (26).

In rural Bangladesh, 84% of households have access to a mobile phone (27). A study in Bangladesh revealed women using Aponjon, a mobile-messaging based service providing information about mother and babies, had higher rates of autonomy in accessing mobile phones, although two-thirds of the women resided in urban areas. mHealth consultations may help address socio-cultural gender norms, and empower women as they were able to discuss personal health issues on the phone with female doctors who are infrequently found in rural communities (27). The need to collect sex aggregated, valid, comprehensive and standardised empowerment data is therefore imperative to achieve not only SDG 5 but to enable the consistent measurement of women's empowerment indices (28, 29).

Objectives and Hypotheses

This study aims to evaluate the impact of a combined nutrition counselling and unconditional cash transfer intervention (Shonjibon Cash and Counselling / SCC Trial), delivered on a mobile platform, on the level of empowerment of women in rural Bangladesh. This study will assess women's experience of empowerment and disempowerment throughout the SCC trial.

For peer review only - http://bmjopen.bmj.com/site/about/guidelines.xhtml

The **primary hypothesis** is that in a community-based cluster randomised control trial of a mobile phone-based nutrition behaviour change communication (BCC) and unconditional cash transfer, given to women in low-income families in rural Bangladesh, women's empowerment will increase as measured by an increase in mean women's empowerment scores (by an average percentage of 20) from the baseline to the end of the 24-month intervention, compared to women in the control arm.

The **secondary hypotheses** are that the mobile phone nutrition BCC and unconditional cash transfer compared to usual programs will: a) increase control over income and economic resources; b) improve input and decision making power in nutrition and health care choices; c) decrease the acceptance and experience of intimate partner violence.

SCC Trial women's empowerment Theory of Change

Empowering women is not an explicit goal of the SCC Trial; however, women gain access to nutrition counselling, cash transfers and a mobile phone; the utilisation of these resources can empower women. We constructed the theory of change to illustrate how the SCC Trial will impact on women's empowerment outcomes. It outlines the barriers, intervention details, short and long term outcomes and impact pathways (Figure 1).

The SCC Trial intervention is delivered on a mobile platform. Women receive a mobile phone with an interactive app – messages, audio and video, quizzes – as well as counselling from the call centre. They also receive cash via BKash mobile banking app. The principal rationale of the theory of change causal pathway to impact is that SCC Trial will lead to an increase in women's empowerment for those women participating in the intervention arm.

The Project-level Women's Empowerment in Agriculture Index (Pro-WEAI)(28) indicators, discussed in detail later, appear in the theory of change across the short and long term outcomes. We will use the six indicators from the Pro-WEAI to calculate the women's empowerment index and include: 1) Control over

For peer review only - http://bmjopen.bmj.com/site/about/guidelines.xhtml

use of income; 2) Input into productive decisions; 3) Autonomy in income; 4) Decision-making power on nutrition and health care; 5) Respect among household members; 6) Attitudes toward IPV.

One of the barriers to nutrition faced in rural Bangladesh is limited access to nutrition and infant and young child feeding (IYCF) information. Women in the SCC Trial intervention arm receive nutrition, IYCF and livelihood BCC delivered on a mobile app and nutrition counselling from the call centre. The short term outcome is woman's acquisition of new BCC/nutrition knowledge, which in turn as a long-term outcome, contributes to increased input into and decision making power about health and nutrition choices for herself and her family (Indicator 4). Improvements in communication also occur (spousal, intrahousehold, community) as new knowledge is discussed and shared, including ways to put the messages into action (Indicator 5). One's ability to make decisions is a measure of empowerment (30). New knowledge can be used as leverage and bargaining power and enhance decision-making abilities across a range of areas such as livelihoods, household expenditure and decisions on productive assets, i.e. what crops to farm (Indicator 2,3,4).

The SCC Trial setting is in one of the poorest regions in Bangladesh. Families face barriers such as food insecurity and lack of financial resources. When a woman is the recipient of a cash transfer, the short term outcome is increased control over the use of income (Indicator 1). Cash transfers, targeted at women, have the potential to improve livelihoods, alleviate poverty, enhance food security and increase women's empowerment (9). Spousal communication is also strengthened as discussion ensues as to how to spend the additional income (Indicator 4). The long term outcomes for women receiving an unconditional cash transfer include a strengthened ability to contribute to and participate in financial decision making. This decision making covers health and nutrition choices, household expenditure, productive assets, livelihood choices (i.e. homestead gardening, buying chickens to raise for eggs, meat and income) (Indicator 1,2,3,4).

For peer review only - http://bmjopen.bmj.com/site/about/guidelines.xhtml

IPV includes behaviour that is physical, psychological, economic, sexual or abusive or controlling in nature. In lower-income settings, women frequently defend IPV against women, more often than men (31). There is evidence that the combination of additional income from cash transfers combined with BCC, can increase women's bargaining power and poverty-related emotional well-being (8). Changing attitudes, from within the family unit, as women participate and learn from intervention activities has the potential to lead to a change in the acceptability of IPV, linked to a woman's right to bodily integrity (32). Women may also experience an increase in respect and self-efficacy and subsequent reduction in acceptance of IPV (Indicator 6).

Figure 1 SCC Trial women's empowerment Theory of Change (adapted from De Silva et al. (33)

Study Design

We will embed this study on women's empowerment within the SCC Trial. The SCC Trial will recruit pregnant women and follow up over 24 months an expected 2,080 mother-child dyads, from recruitment in early pregnancy until the child is 18 months of age. The intervention will run for 36 months, depending on the time of enrolment and gestation, and age of the child.

Methods

This study will use a mixed-methods approach with data gathered on women's empowerment from participants in the SCC Trial. This protocol paper will adhere to the SPIRIT and Tidier guidelines for clinical trial protocols (34-36) to ensure rigour in protocol content.

Study setting

We will conduct the study in two subdistricts of Sirajganj; Ullapara and Kamarkhanda, in northern Bangladesh (Figure 2). Employment in Sirajganj is predominantly in agriculture (51 %) with 25 % working in services and 23% in industry (37).

For peer review only - http://bmjopen.bmj.com/site/about/guidelines.xhtml

Figure 2: Sirajganj, Bangladesh SCC Study site

Eligibility criteria

All married women between 15-49 years, that are permanent residents of the study area and provide consent to participate and test positive with pregnancy urine test kit (Excel®) (whose gestational age is ≤90 days) are eligible to participate in SCC Trial.

Intervention: Shonjibon[1] Cash and Counselling Trial

A separate protocol paper provides details of the design of the SCC Trial. Briefly, the SCC Trial aims to assess the effectiveness of nutrition behaviour change communication (delivered via call centre counselling and specifically tailored mobile app) combined with unconditional cash transfers in reducing the prevalence of stunting (length-for-age < -2 Z) in children at 24 months. We will use a longitudinal cluster randomised controlled trial to determine the effectiveness of unconditional cash transfers and mobile behaviour change communications (BCC) to reduce child undernutrition in Sirajganj district in northern Bangladesh, one of the poorest regions in the country according to the World Food Program Poverty Maps (38). Both quantitative and qualitative approaches will be employed to address the study objectives.

The SCC Trial comprises of three types of intervention: 1) nutrition BCC delivered on a specially tailored app on a smartphone (audio, video, and animation), 2) direct nutrition counselling from a call centre and 3) unconditional cash transfer of 1000 Taka (USD 12.50) received monthly via BKash mobile banking app. The control arm will receive a mobile phone and the current Government of Bangladesh health and nutrition services. The participants in the control arm receive a mobile phone as the trial is interested in

[1] [1]Shonjibon stands for Shustho Notun Jibon in Bangla, translated, it means "Healthy New Life"

For peer review only - http://bmjopen.bmj.com/site/about/guidelines.xhtml

the effects of the messages, nutrition counselling and cash transfer delivered on a mobile platform, as opposed to the non-specific effects of a mobile phone.

Study outcomes

The primary study outcome will be the difference in women's empowerment scores in the intervention arm as compared to the control arm of the SCC Trial. Secondary study outcomes will include 1) an increase in control over income and economic resources; 2) improved input and decision making power in nutrition and health care choices; 3) experience a decrease in intimate partner violence.

Outcome measurements

The SCC trial will collect data across the main module of the Pro-WEAI (12 indicators) as well as the additional health and Pro-WEAI Nutrition and Health module. However, 6 of the Pro-WEAI indicators are likely to be directly impacted by the SCC trial, therefore, we will develop a composite women's empowerment indicator based on the following: 1) control over the use of income; 2) input in production decisions; 3) autonomy in income; 4) decision making power about women and children's health and nutrition; 5) respect among household members and 6) attitudes toward IPV (Appendix Table 1). We will also measure women's and family member's perceived and lived experiences related to women empowerment qualitatively.

Autonomy and control over use of income and input into productive decisions

We will measure autonomy and control over the use of income by asking questions from Pro-WEAI; these questions are significant as the SCC Trial gives an unconditional cash transfer to the woman. Qualitative interviews will explore local perceptions of intrahousehold harmony and

For peer review only - http://bmjopen.bmj.com/site/about/guidelines.xhtml

communication skills, issues related to withdrawing the cash, and perceptions and experience of control and expenditure of the money.

Decision-making ability about health and nutrition choices

We will examine responses from the Pro-WEAI Nutrition and Health Module regarding confidence and participation in decisions about nutrition and infant feeding, as well as a range of issues affecting women and children such as whether to consult a doctor when ill to dietary choices when pregnant.

Intimate Partner Violence

Previous research in Bangladesh has found that cash transfers and behaviour change communication can reduce IPV (8). We will measure a woman's attitude toward IPV using the Pro-WEAI as well as the direct experience of IPV using the questions from the Violence Against Woman and Girls 2015 Survey (39, 40). We will also conduct in-depth interviews to explore the woman's attitudes and perceptions of IPV qualitatively.

Participant recruitment and timeline

A household census in the study area will record the names and contact details of all women that agree to participate, and we will enter their details into an electronic system. The system will generate for each participant with a unique ID number and quick response (QR) code. SCC surveillance workers will then conduct door-to-door visits each month to identify women that have missed two menstrual periods in a row. The woman will then undertake a pregnancy test (using a urine test kit (Excel®). We will invite women who test positive for pregnancy to participate in the study with appropriate informed written consent. Based on experience with our pilot study, we anticipate 95% of the mothers will consent. Qualitative and quantitative data will be gathered at the start of the intervention and at endline (Appendix Table 2).

Allocation of clusters

For peer review only - http://bmjopen.bmj.com/site/about/guidelines.xhtml

Our study site has over 1100 villages, which would be adequate to cover the anticipated sample size. Each cluster encompasses 2-4 villages and consists of ~1000 households and ~4500 population. We will use a fixed randomization scheme to assign treatments to eligible clusters, with a uniform allocation ratio of treatments, stratified by Union. The sample size will consist of 104 clusters, total women (N = 2080) allocated to two parallel groups (n = 1040) with 20 mother-infant dyads per cluster. Each cluster consists of 1-4 adjacent villages with a minimum population of 600 households per cluster with 454 villages in our study area for clustering. A subsample of the households from a purposively selected subset of clusters will be chosen for qualitative data.

The intervention and control groups will receive similar maternal and child health and nutrition services from the government, and the critical difference will be the nutrition behaviour change communications with cash transfers on a mobile platform received by women in the intervention arm. The study design will control for potential observed and unobserved confounding factors as there will be an adequate number of clusters randomly allocated to the treatment groups. The geographic separation of clusters will limit the contamination of the intervention arm. We will use Stata® software to generate the random allocation sequence. We are unable to mask the treatment arm due to the nature of the intervention.

Data Collection methods

The SCC Trial will collect key household data and nutrition indices, while this study collects data on women's empowerment using the Pro-WEAI, whose focus is measuring agency for which there are limited if any standardised measures (28). We will include the Pro-WEAI nutrition and health module and supplementary questions regarding intimate partner violence. The qualitative component will be tailored to context-specific issues and guided by formative research.

Quantitative data

For peer review only - http://bmjopen.bmj.com/site/about/guidelines.xhtml

311 We will collect the women's empowerment indicators in household surveys at the baseline and the endline of the 24-month study in the intervention and non-intervention arms. We will pre-test all questions before the commencement of the study. We also ask a short version of Marlowe-Crowne's validated social desirability questions (41) (Appendix Table 3). The questionnaire assesses whether social desirability bias or the tendency of respondents to answer questions in a way viewed favourably by the research team has influenced the data collected. We will train the field researchers in detecting social desirability bias in qualitative data and limit bias by properly introducing the study to the respondents, building rapport and asking context-specific probing questions following the recent framework described by Bergen and Labonté (42). We will follow the process throughout the research.

320 Qualitative data

321 The study uses qualitative analysis to elicit a local understanding of women's empowerment and the linkages between the SCC Trial and women's empowerment outcomes. We will generate qualitative data on women and men's perceived and experienced change in empowerment through longitudinal qualitative semi-structured in-depth interviews. We will also interview key family members as suggested by the women, for example, husband or mother-in-law.

326 We will explore the myriad of ways that women and men perceive and describe empowerment in their communities. A purposive sample will be interviewed approximately two months after the start of the intervention and at the endline of the SCC Trial. Due to the highly sensitive nature of the topics, a separate team of interviewers, from a different Upazila, will be trained to collect data on women's empowerment and intimate partner violence. We will train the team in line with the ethical and safety procedures as per the World Health Organization's guidelines (43). The training will cover measures such as changing topics and asking a decoy nutrition question if anyone comes into the interview space and having details of referral and support services for women affected by or experiencing IPV.

For peer review only - http://bmjopen.bmj.com/site/about/guidelines.xhtml

334 ***Pro-WEAI***

335 The Pro-WEAI measures the empowerment of women, agency and inclusion in the agriculture sector (28). 336 It is a standardised, survey-based internationally validated index, developed to adapt to project-specific 337 contexts and has been piloted in Bangladesh (24, 28). This study uses Kabeer's conceptual framework of 338 empowerment focuses on the ability of individuals to make choices, ''a process of change during which 339 those who have been denied the ability to make choices acquire such an ability'' (44, 45). Agency or the 340 ability of the individual to make considered choices is at the centre of the framework and revolves around 341 interrelated dimensions of resources, agency and achievements. This framework underpins the Pro-WEAI 342 (28). This study will collect data from women, across twelve indicators that are equally weighted (Figure 343 3), with the addition of the Project-specific Nutrition and Health module.

344 *Figure 3: Pro-WEAI 3 Domains of Empowerment and 12 Indicators (28)*

345 ***Intimate Partner Violence***

346 The Pro-WEAI includes questions on a woman's attitude to intimate partner violence (IPV). We will collect 347 additional data about personal experience and behaviour specific questions regarding IPV, as opposed to 348 her perceptions thereof. We took these questions from the WHO Violence Against Women and Girls 349 Survey (Bangladesh 2015) (40). We will also ask questions relating to cyberviolence (46).

350 ***Health and Nutrition***

351 The Pro-WEAI offers the option of adding project-specific modules, and for this study, we will add the 352 Nutrition and Health Module. This module asks questions about decision making about nutrition and 353 health indices for women and children.

354 **Data collection and management**

For peer review only - http://bmjopen.bmj.com/site/about/guidelines.xhtml

Intervention and evaluation staff will be separate to and not know the trial hypothesis to minimise assessment bias. Android Tablets will be used in the field to enable interactive data collection. During each evaluation interview, the data collectors will be guided and navigated by the software. As soon as the field staff complete each interview, they will upload the data to the server at icddr,b. The data will be retained on the tablet and copied/merged with files at icddr,b., with android tabs being synchronised with the server daily. We will systematically scrutinise the captured data and address any discrepancies as they occur. Qualitative narrative data will be captured in digital audio recorders and stored in the server from where we will check for quality and improvement for future interviews.

Data Analysis

We will record all interviews using digital audio recording devices. Interviews will be transcribed verbatim in Bangla by the research team at icddr,b. We will maintain quality control, by checking the transcripts at random intervals, to ensure the quality and accuracy of transcriptions. Data will be coded using thematic analysis approach (47). We will use NVivo software to organise, code, categorise and compile the data. Qualitative methods offer an effective way of describing in-depth, complex and varied perceptions of empowerment within the local context. They will also aid with the interpretation of individual indicators within the quantitative data. Data triangulation by using different sources of data will add strength to the analysis (48).

Data safety and monitoring board

An independent data and safety monitoring board will be formed to assess the completeness and quality of data. Any unintended effects of the trial will be reported to the board.

Dissemination plan

For peer review only

For peer review only - http://bmjopen.bmj.com/site/about/guidelines.xhtml

Lessons learned from this study will be shared in Bangladesh with dissemination sessions in-country. Internationally, key findings will be shared with stakeholders, results will be presented at conferences, and published in international peer-reviewed journals.

Access to data

The SCC investigators will have access to all data and the right to analyse and publish data. Datasets will be shared after all personally identifiable information has been removed and to keep the identification of study subjects in confidence.

Ethics approval and consent

We have obtained approval for the SCC Trial from the Ethical Review Committee (ERC) of the International Centre for Diarrhoeal Disease Research (icddr,b) – (Ref. PR 17106), and the Human Research Ethics Committees (HRECs) at The University of Sydney (Ref: 2019/840).

We will train the women on the use of smartphones as well as the nutrition counselling application. We will store the data obtained on the use of the application with access to limited authorised project staff. Trained field staff will educate the women about the safety procedures for receipt and use of money. We will record responses anonymously and by identification number. We will maintain the confidentiality, privacy, and anonymity of the participants.

Project field workers will explain the nature, purpose, benefits, risks, and process of the trial to potential participants as they register themselves in the surveillance system if they meet the inclusion criteria. We will train them to obtain written informed consent (in Bangla) from all intervention participants. Participation in the research will be voluntary, and the respondent will have every right to withdraw anytime throughout the programme without any obligation, loss or penalty. We will explain the participant's rights before collecting any data.

For peer review only

For peer review only - http://bmjopen.bmj.com/site/about/guidelines.xhtml

Discussion

The main goal of this study is to evaluate the impact of a combined nutrition counselling and unconditional cash transfer intervention, delivered on a mobile platform, on women's empowerment in rural Bangladesh. We recognise that women's empowerment is an essential goal in global public health and the key to sustainable development. It is therefore crucial, that in complex and large scale nutrition interventions, such as the SCC Trial, that we measure intermediary outcomes along pathways of impact such as the domains of women's empowerment. This study has several strengths; firstly, it is embedded within a cluster RCT that aims to assess the effectiveness of a nutrition behaviour change communication combined with unconditional cash transfers in reducing the prevalence of stunting. Another strength lies in the use of a mixed-methods approach – obtaining qualitative and quantitative data. We gain further advantage from the use of tools tailored specifically for this intervention; tools that are based on formative research, wide-ranging literature review and piloted in Bangladesh, providing accurate, comprehensive and appropriate methodology.

The Pro-WEAI is designed to assess the impact evaluation of agricultural development projects (49). The SCC Trial is not an agricultural intervention, however, women in Bangladesh characteristically do postharvest activities and processing yet do not classify themselves as agricultural workers. It is in this context that we are using the index, to survey women in Bangladesh, whose livelihoods are bound to the agricultural sector (50). Alkire (2018) describes the Pro-WEAI as an "information platform" that is applicable in broader contexts other than exclusively agricultural interventions (51). The core elements of the SCC women's empowerment index, a customised tool, will provide translatable results – using measures and indices that allow global comparisons.

When the Pro-WEAI is used in full – women and men are interviewed - this enables the calculation of the Gender Parity Index, which measures empowerment and disempowerment within the household. The

For peer review only - http://bmjopen.bmj.com/site/about/guidelines.xhtml

limitation of not interviewing men is that we may not capture the sense of intrahousehold inequality - are the women at least as empowered as the men in their households? We will account for this by ensuring that qualitative exploration delves into the extent to which the disempowerment observed is attributable to gender norms versus other causes. The SCC Trial does not directly focus on women's empowerment nor contest existing gender norms or patriarchal power structures. The study participants - the recipients of the cash transfer, nutrition counselling and smartphone - are, however, women. This study will identify where gaps in empowerment occur, highlight key areas of disempowerment and which domains help facilitate or hinder nutrition outcomes.

Funding

The Shonjibon Cash and Counselling Trial is funded by the National Health and Medical Research Council of Australia GNT 1120507. EKK received seed funding for a field visit from the University of Sydney Faculty of Medicine and Health. The funders do not have any role in the study design, data collection, and interpretation of data.

Trial registration:

- The Shonjibon Cash and Counselling Trial is registered with the Australian New Zealand Clinical Trials Registry (ANZCTR) - The registration ID is ACTRN 12618001975280.

Abbreviations

- BCC behaviour change communication
- FGD focus group discussions
- icddr,b International Centre for Diarrhoeal Disease Research
- IDI in-depth-interview (IDI)
- IFPRI International Food Policy Research Institute

For peer review only

- IPV intimate partner violence
- IYCF infant young child feeding
- NHMRC National Health and Medical Research Council of Australia
- Pro-WEAI Project-Level Women's Empowerment in Agriculture Index
- SCC Trial Shonjibon Cash and Counselling Trial

Author's contributions

Elizabeth K Kirkwood drafted the paper, designed the study tools and main conceptual ideas. Ashraful Alam and Michael Dibley made continuous contributions and supervised in the design of the study and throughout the writing. John Hoddinott, Kingsley Agho, Tanvir Huda, Tazeen Tahsina, Nazia Ali and Tracey Lea-Laba reviewed the draft.

Acknowledgements

We gratefully acknowledge the team at the International Food Policy Research Institute for their assistance and sharing the Project-Level Women's Empowerment in Agriculture Index. I would also like to thank the seminar attendees at the Sydney School of Public Health's Global Health and Nutrition Research Collaboration for providing valuable feedback at my presentation on the tools and methodology used in this paper.

Competing Interests

The authors declare that they do not have any competing interests.

For peer review only

For peer review only - http://bmjopen.bmj.com/site/about/guidelines.xhtml

References

1. Alsop R, Bertelsen MF, Holland J. Empowerment in practice - from analysis to implementation Washington, DC 20433 USA; 2006.
2. Department of Economic and Social Affairs UPD. Guidelines on Women's Empowerment. New York, USA; 2010.
3. Heise L, Greene ME, Opper N, Stavropoulou M, Harper C, Nascimento M, et al. Gender inequality and restrictive gender norms: framing the challenges to health. The Lancet. 2019;393(10189):2440-54.
4. Transforming Our World: The 2030 Agenda for Sustainable Development. . United Nations, New York, USA.: United Nations; 2015. Contract No.: 09/10/2018.
5. The Future We Want. Rio + 20, Rio de Janeiro, Brazil: United Nations; 2012.
6. Kabeer N. The Power to Choose: Bangladeshi Women and Labour Market Decisions in London and Dhaka: Verso Books; 2000.
7. Global and regional estimates of violence against women: prevalence and health effects of intimate partner violence and non-partner sexual violence. Geneva: World Health Organization; 2013.
8. Roy S, Hidrobo M, Hoddinott J, Ahmed A. Transfers, Behavior Change Communication, and Intimate Partner Violence: Postprogram Evidence from Rural Bangladesh. The Review of Economics and Statistics. 2019;101(5):865-77.
9. Ruel MT, Alderman H. Nutrition-sensitive interventions and programmes: how can they help to accelerate progress in improving maternal and child nutrition? The Lancet. 2013;382(9891):536-51.
10. Kim JC, Watts CH, Hargreaves JR, Ndhlovu LX, Phetla G, Morison LA, et al. Understanding the impact of a microfinance-based intervention on women's empowerment and the reduction of intimate partner violence in South Africa. Am J Public Health. 2007;97(10):1794-802.
11. Kabeer N. Women’s Empowerment, Development Interventions and the Management of Information Flows. Institute of Development Studies. 2010;41(6).
12. Malhotra A, Schuler SR. Women’s Empowerment as a Variable in International Development. In: Bank TW, editor. Measuring Empowerment: Cross Disciplinary Perspectives2005. p. 71- 88.
13. The State of Food Security and Nutrition in the World 2018. Building climate resilience for food security and nutrition. . Rome, FAO: FAO, IFAD, UNICEF, WFP and WHO; 2018.
14. Patalagsa MA, Schreinemachers P, Begum S, Begum S. Sowing seeds of empowerment: effect of women’s home garden training in Bangladesh. Agriculture & Food Security. 2015;4(1):24.
15. Turning promises into action: Gender equality in the 2030 Agenda for Sustainable Development. New York, United Nations Women: UN Women; 2018.
16. Gillespie S, Hoddinott J, Nisbett N, Arifeen S, van den Bold M. Evidence to Action: Highlights From Transform Nutrition Research (2012-2017). Food Nutr Bull. 2018;39(3):335-60.
17. Heymann J, Levy JK, Bose B, Ríos-Salas V, Mekonen Y, Swaminathan H, et al. Improving health with programmatic, legal, and policy approaches to reduce gender inequality and change restrictive gender norms. The Lancet. 2019;393(10190):2522-34.
18. Cunningham K, Ruel MT, Ferguson E, Uauy R. Women's empowerment and child nutritional status in South Asia: a synthesis of the literature. Maternal & Child Nutrition. 2015;11(1):1-19.
19. Jennings L, Gagliardi L. Influence of mhealth interventions on gender relations in developing countries: a systematic literature review. International Journal for Equity in Health. 2013;12(1):85.
20. Campos APdlO. Empowering rural women through social protection. Rome: FAO - Food and Agriculture Organization of the United Nations

2015.

For peer review only - http://bmjopen.bmj.com/site/about/guidelines.xhtml

21. Headey D, Hoddinott J, Ali D, Tesfaye R, Dereje M. The Other Asian Enigma: Explaining the Rapid Reduction of Undernutrition in Bangladesh. World Development. 2015;66:749-61.
22. Employment in agriculture (% of total employment) (modeled ILO estimate) [Internet]. 2018 [cited 13/05/2019]. Available from: https://data.worldbank.org/indicator/SL.AGR.EMPL.ZS?locations=BD.
23. Women Farmers of Bangladesh. Food and Agriculture organization of the United Nations; 2016.
24. Alkire S, Meinzen-Dick R, Peterman A, Quisumbing A, Seymour G, Vaz A. The Women's Empowerment in Agriculture Index. World Development. 2013;52:71-91.
25. Sraboni E, Malapit HJ, Quisumbing AR, Ahmed AU. Women's Empowerment in Agriculture: What Role for Food Security in Bangladesh? World Development. 2014;61:11-52.
26. Huda TM, Alam A, Tahsina T, Hasan MM, Khan J, Rahman MM, et al. Mobile-Based Nutrition Counseling and Unconditional Cash Transfers for Improving Maternal and Child Nutrition in Bangladesh: Pilot Study. JMIR mHealth and uHealth. 2018;6(7):e156-e.
27. Alam M, Banwell C, Olsen A, Lokuge K. Patients' and Doctors' Perceptions of a Mobile Phone–Based Consultation Service for Maternal, Neonatal, and Infant Health Care in Bangladesh: A Mixed-Methods Study. JMIR Mhealth Uhealth. 2019;7(4):e11842.
28. Malapit HJ, Quisumbing AR, Meinzen-Dick RS, Seymour G, Martinez EM, Heckert J, et al. Development of the Project-Level Women's Empowerment in Agriculture Index (Pro-WEAI). IFPRI; 2019 January 18, 2019.
29. Weber AM, Cislaghi B, Meausoone V, Abdalla S, Mejía-Guevara I, Loftus P, et al. Gender norms and health: insights from global survey data. The Lancet. 2019;393(10189):2455-68.
30. Seymour G, Peterman A. Context and measurement: An analysis of the relationship between intrahousehold decision making and autonomy. World Development. 2018;111:97-112.
31. Uthman OA, Lawoko S, Moradi T. Sex disparities in attitudes towards intimate partner violence against women in sub-Saharan Africa: a socio-ecological analysis. BMC Public Health. 2010;10(1):223.
32. Malapit H, Quisumbing A, Meinzen-Dick R, Seymour G, Martinez EM, Heckert J, et al. Development of the project-level Women's Empowerment in Agriculture Index (pro-WEAI). World Development. 2019;122:675-92.
33. De Silva MJ, Breuer E, Lee L, Asher L, Chowdhary N, Lund C, et al. Theory of Change: a theory-driven approach to enhance the Medical Research Council's framework for complex interventions. Trials. 2014;15(1):267.
34. Hoffmann Tammy C GPP, Boutron Isabelle, Milne Ruairidh, Perera Rafael, Moher David, Virginia Barbour, Marie Johnston, Sarah E. Lamb, Mary Dixon-Woods, Jeremy C. Wyatt. Better reporting of interventions: template for intervention description and replication (TIDieR) checklist and guide. BMJ. 2014:348:g1687.
35. Chan A-W, Tetzlaff JM, Altman DG, Laupacis A, Gøtzsche PC, Krleža-Jerić K, et al. SPIRIT 2013 Statement: Defining Standard Protocol Items for Clinical Trials. Annals of Internal Medicine. 2013;158(3):200-7.
36. Chan A-W, Tetzlaff JM, Gøtzsche PC, Altman DG, Mann H, Berlin JA, et al. SPIRIT 2013 explanation and elaboration: guidance for protocols of clinical trials. BMJ : British Medical Journal. 2013;346:e7586.
37. Bangladesh Interactive Poverty Maps: The World Bank; 2016 [Available from: http://www.worldbank.org/en/data/interactive/2016/11/10/bangladesh-poverty-maps.
38. Poverty Maps of Bangladesh 2010. World Food Programme (WFP); 2010 2010.
39. García-Moreno C, Jansen HAFM, Ellsberg M, Heise L, Watts C. WHO multi-country study on women's health and domestic violence against women. Geneva: World Health Organization; 2005.
40. Bernard T, Doss C, Hidrobo M, Hoel J, Kieran C. Ask me why: Patterns of intrahousehold decision-making. World Development. 2020;125:104671.

For peer review only - http://bmjopen.bmj.com/site/about/guidelines.xhtml

41. Reynolds WM. Development of reliable and valid short forms of the marlowe-crowne social desirability scale. Journal of Clinical Psychology. 1982;38(1):119-25.
42. Bergen N, Labonté R. “Everything Is Perfect, and We Have No Problems”: Detecting and Limiting Social Desirability Bias in Qualitative Research. Qualitative Health Research. 2020;30(5):783-92.
43. Ellsberg M, Heise L. Researching Violence Against Women: A Practical Guide for Researchers and Activists. Washington DC, United States: World Health Organization, PATH; 2005.
44. Kabeer N. Resources, Agency, Achievements: Reflections on the measurement of Women's Empowerment. Development and Change. 1999;30(3):435-64.
45. Kabeer N. Gender equality and women's empowerment: A critical analysis of the third millennium development goal 1. Gender & Development. 2005;13(1):13-24.
46. Navid RT, Samuels F, Le MAsson V, Talukder A, Gupta T, Yount KM. Understanding intimate partner violence in rural Bangladesh: Prevention and response. London: ODI, Emory University, icddr,b; 2017 May 2017.
47. Braun V, Clarke V. Using thematic analysis in psychology. Qualitative Research in Psychology. 2006;3(2):77-101.
48. Guion L, Diehl D, McDonald D. Triangulation: Establishing the Validity of Qualitative Studies. University of Florida, The Institute of Food and Agricultural Sciences (IFAS) 2011.
49. Yount KM, Cheong YF, Maxwell L, Heckert J, Martinez EM, Seymour G. Measurement Properties of the Project-Level Women’s Empowerment in Agriculture Index. International Food Policy Research Institute; 2019.
50. WEAI FAQs: International Food Policy Research Institute 2018 [Available from: https://weai.ifpri.info/weai-resource-center/faqs-2/.
51. Alkire S. Multidimensional Poverty Measures as Relevant Policy Tools. Oxford Poverty & Human Development Initiative 2018.

For peer review only - http://bmjopen.bmj.com/site/about/guidelines.xhtml

Appendix

Table 1: SCC women's empowerment summary index components

**Shonjibon Trial resources**	**Pathway**	**Outcome variable / index components**
Mobile-based (app and call centre) **BCC on Nutrition**, IYCF and livelihoods<br><br>unconditional **cash transfer** delivered on BKash mobile banking app<br><br>**Mobile phone** (with a data package, SMS and talk time)	Instrumental Agency (Power to)	1. Control over use of income (G2)<br>2. Input in production decisions (G2)
	Intrinsic agency (power within)	3. Autonomy in income (G8(a))<br>4. Decision-making power on nutrition and health care (X)<br>5. Respect among household members (G7)<br>6. Attitudes toward IPV (G9)

For peer review only

Table 2: Data collection timeline

**Methods**	**Participants**	**timeline**
Semi-structured in-depth interview at the household level		
In-depth interviews with women	1) Pregnant women<br>2) Mothers of children <1 month<br>3) Mothers 1-5 months<br>4) Mothers of children 6 – 59 months	➢ At the start of SCC Trial<br>➢ Endline
In-depth interviews with family members	1) Mother or mother-in-law of women<br>2) Husband of women	➢ At the start of SCC Trial<br>➢ Endline

For peer review only - http://bmjopen.bmj.com/site/about/guidelines.xhtml

	3) Other key family members as directed by the woman interviewed	
Pro-WEAI Survey questions		
Survey questionnaires	Female intervention recipients	➢ Baseline<br>➢ Endline

Table 3: Social Desirability Bias

**Directions: Read each item and decide whether it is true (T) or false (F) for you.**			
		**True**	**False**
1	It is sometimes hard for me to go on with my work if I am not encouraged.		
2	I sometimes feel resentful when I don't get my way		
3	On a few occasions, I have given up doing something because I thought too little of my ability		
4	There have been times when I felt like rebelling against people in authority even though I knew they were right		
5	No matter who I'm talking to, I'm always a good listener		
6	There have been occasions when I took advantage of someone		
7	I'm always willing to admit it when I make a mistake		
8	I sometimes try to get even rather than forgive and forget		
9	I am always courteous, even to people who are disagreeable		
10	I have never been irked when people expressed ideas very different from my own		
11	There have been times when I was quite jealous of the good fortune of others		
12	I am sometimes irritated by people who ask favours of me		
13	I have never deliberately said something that hurt someone's feelings		

For peer review only

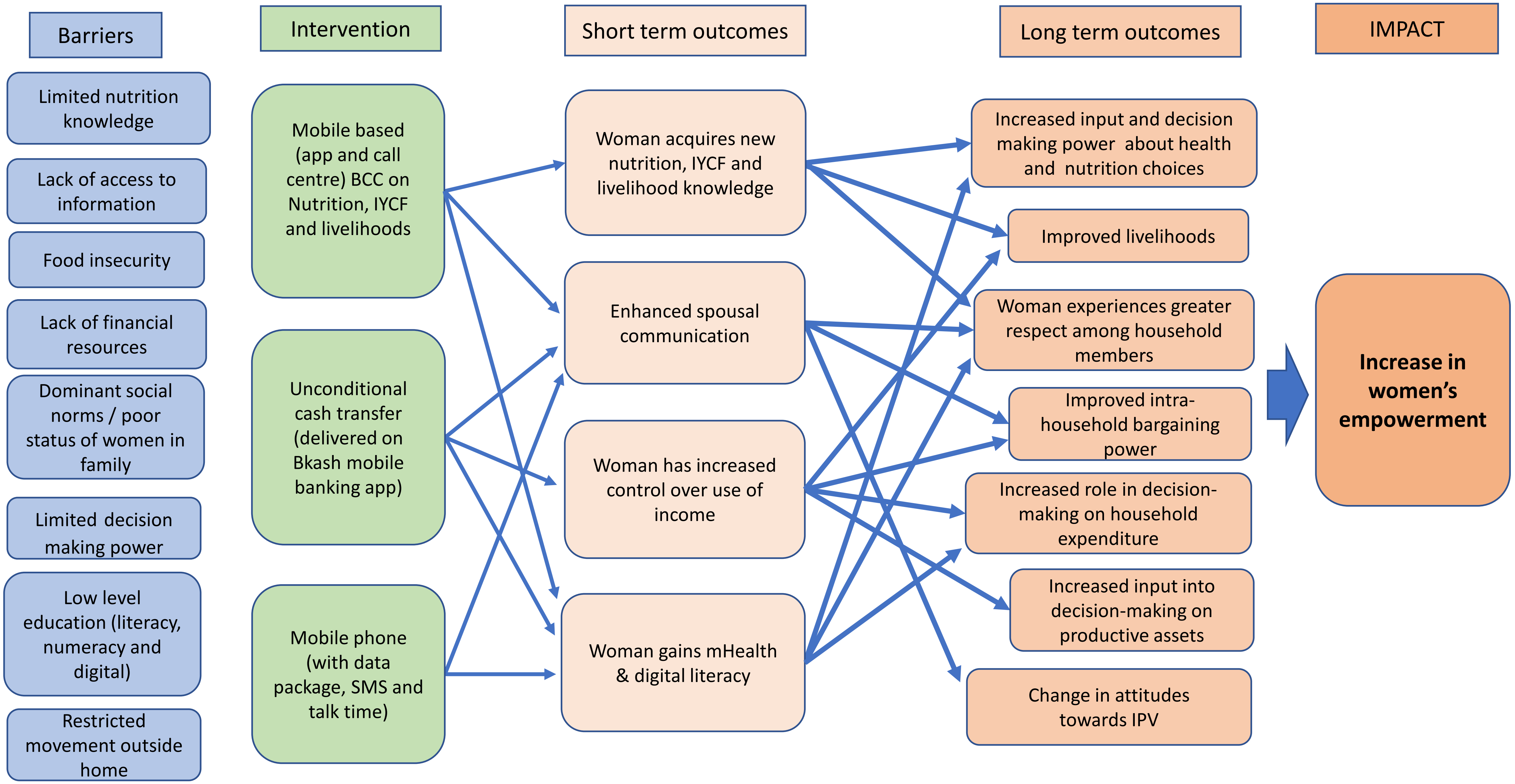
Barriers
Limited nutrition knowledge
Lack of access to information
Food insecurity
Lack of financial resources
Dominant social norms / poor status of women in family
Limited decision making power
Low level education (literacy, numeracy and digital)
Restricted movement outside home
Intervention
Mobile based (app and call centre) BCC on Nutrition, IYCF and livelihoods
Unconditional cash transfer (delivered on Bkash mobile banking app)
Mobile phone (with data package, SMS and talk time)
Short term outcomes
Woman acquires new nutrition, IYCF and livelihood knowledge
Enhanced spousal communication
Woman has increased control over use of income
Woman gains mHealth & digital literacy
Long term outcomes
Increased input and decision making power about health and nutrition choices
Improved livelihoods
Woman experiences greater respect among household members
Improved intra-household bargaining power
Increased role in decision-making on household expenditure
Increased input into decision-making on productive assets
Change in attitudes towards IPV
IMPACT
Increase in women's empowerment

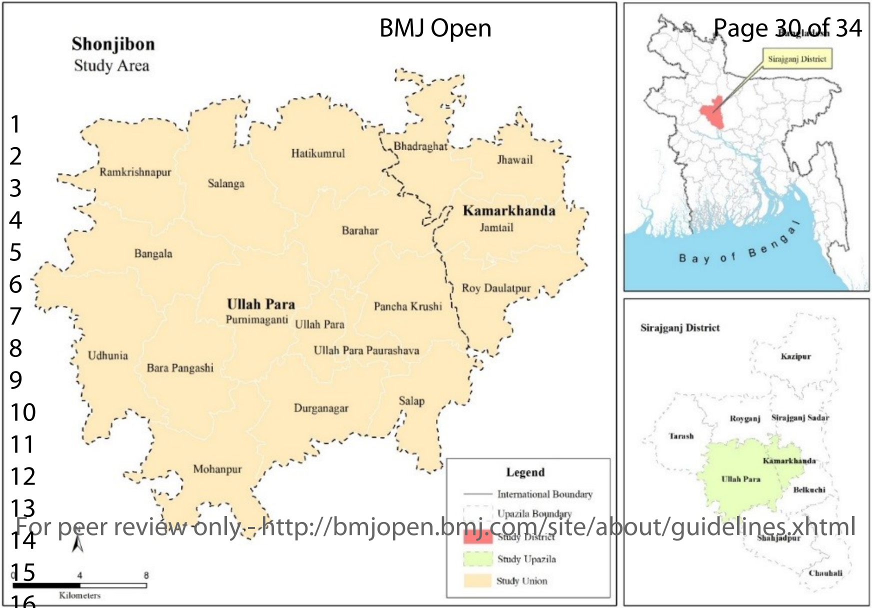

For peer review only - http://bmjopen.bmj.com/site/about/guidelines.xhtml

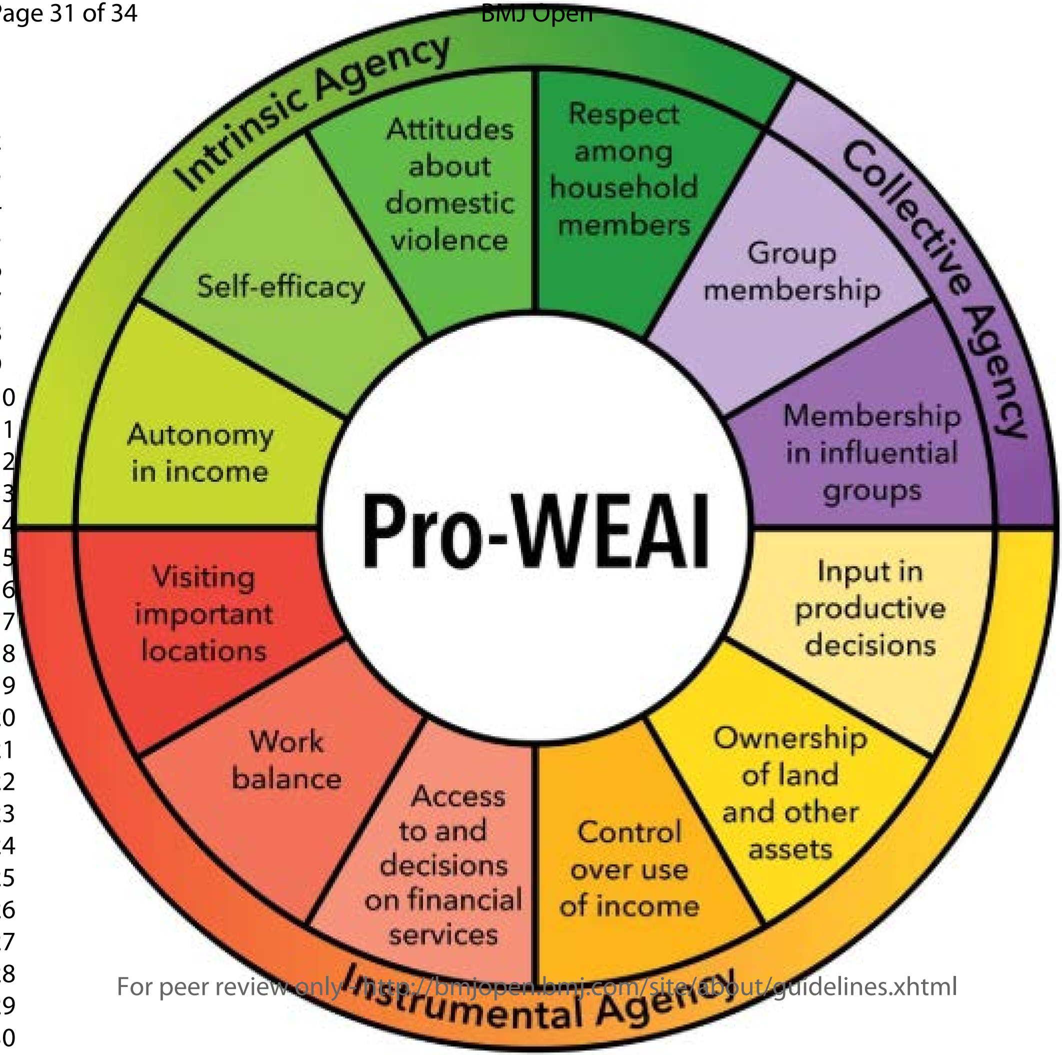

For peer review only - http://bmjopen.bmj.com/site/about/guidelines.xhtml

STANDARD PROTOCOL ITEMS: RECOMMENDATIONS FOR INTERVENTIONAL TRIALS

SPIRIT 2013 Checklist: Recommended items to address in a clinical trial protocol and related documents*

Section/item	Item No	Description	Addressed on page number
**Administrative information**			
Title	1	Descriptive title identifying the study design, population, interventions, and, if applicable, trial acronym	___1________
Trial registration	2a	Trial identifier and registry name. If not yet registered, name of intended registry	___20________
	2b	All items from the World Health Organization Trial Registration Data Set	_____________
Protocol version	3	Date and version identifier	_____________
Funding	4	Sources and types of financial, material, and other support	___20_______
Roles and responsibilities	5a	Names, affiliations, and roles of protocol contributors	___1 and 21_
	5b	Name and contact information for the trial sponsor	______________
	5c	Role of study sponsor and funders, if any, in study design; collection, management, analysis, and interpretation of data; writing of the report; and the decision to submit the report for publication, including whether they will have ultimate authority over any of these activities	____22______
	5d	Composition, roles, and responsibilities of the coordinating centre, steering committee, endpoint adjudication committee, data management team, and other individuals or groups overseeing the trial, if applicable (see Item 21a for data monitoring committee)	______________

For peer review only - http://bmjopen.bmj.com/site/about/guidelines.xhtml

Introduction

Background and rationale	6a	Description of research question and justification for undertaking the trial, including summary of relevant studies (published and unpublished) examining benefits and harms for each intervention	____4________
	6b	Explanation for choice of comparators	______________
Objectives	7	Specific objectives or hypotheses	____7 & 8_____
Trial design	8	Description of trial design including type of trial (eg, parallel group, crossover, factorial, single group), allocation ratio, and framework (eg, superiority, equivalence, noninferiority, exploratory)	____13 & 14__

Methods: Participants, interventions, and outcomes

Study setting	9	Description of study settings (eg, community clinic, academic hospital) and list of countries where data will be collected. Reference to where list of study sites can be obtained	____10_________
Eligibility criteria	10	Inclusion and exclusion criteria for participants. If applicable, eligibility criteria for study centres and individuals who will perform the interventions (eg, surgeons, psychotherapists)	____11_________
Interventions	11a	Interventions for each group with sufficient detail to allow replication, including how and when they will be administered	_____14_______
	11b	Criteria for discontinuing or modifying allocated interventions for a given trial participant (eg, drug dose change in response to harms, participant request, or improving/worsening disease)	______________
	11c	Strategies to improve adherence to intervention protocols, and any procedures for monitoring adherence (eg, drug tablet return, laboratory tests)	______________
	11d	Relevant concomitant care and interventions that are permitted or prohibited during the trial	______________
Outcomes	12	Primary, secondary, and other outcomes, including the specific measurement variable (eg, systolic blood pressure), analysis metric (eg, change from baseline, final value, time to event), method of aggregation (eg, median, proportion), and time point for each outcome. Explanation of the clinical relevance of chosen efficacy and harm outcomes is strongly recommended	____12_______
Participant timeline	13	Time schedule of enrolment, interventions (including any run-ins and washouts), assessments, and visits for participants. A schematic diagram is highly recommended (see Figure)	____13________

For peer review only - http://bmjopen.bmj.com/site/about/guidelines.xhtml

Sample size	14	Estimated number of participants needed to achieve study objectives and how it was determined, including clinical and statistical assumptions supporting any sample size calculations	____14_________
Recruitment	15	Strategies for achieving adequate participant enrolment to reach target sample size	____14_________

Methods: Assignment of interventions (for controlled trials)

Allocation:

Sequence generation	16a	Method of generating the allocation sequence (eg, computer-generated random numbers), and list of any factors for stratification. To reduce predictability of a random sequence, details of any planned restriction (eg, blocking) should be provided in a separate document that is unavailable to those who enrol participants or assign interventions	___14__________
Allocation concealment mechanism	16b	Mechanism of implementing the allocation sequence (eg, central telephone; sequentially numbered, opaque, sealed envelopes), describing any steps to conceal the sequence until interventions are assigned	___14_________
Implementation	16c	Who will generate the allocation sequence, who will enrol participants, and who will assign participants to interventions	___14__________
Blinding (masking)	17a	Who will be blinded after assignment to interventions (eg, trial participants, care providers, outcome assessors, data analysts), and how	___14__________
	17b	If blinded, circumstances under which unblinding is permissible, and procedure for revealing a participant's allocated intervention during the trial	_____________

Methods: Data collection, management, and analysis

Data collection methods	18a	Plans for assessment and collection of outcome, baseline, and other trial data, including any related processes to promote data quality (eg, duplicate measurements, training of assessors) and a description of study instruments (eg, questionnaires, laboratory tests) along with their reliability and validity, if known. Reference to where data collection forms can be found, if not in the protocol	____14_________
	18b	Plans to promote participant retention and complete follow-up, including list of any outcome data to be collected for participants who discontinue or deviate from intervention protocols	_____________

Data management	19	Plans for data entry, coding, security, and storage, including any related processes to promote data quality (eg, double data entry; range checks for data values). Reference to where details of data management procedures can be found, if not in the protocol	___17_________
Statistical methods	20a	Statistical methods for analysing primary and secondary outcomes. Reference to where other details of the statistical analysis plan can be found, if not in the protocol	_____17_________
	20b	Methods for any additional analyses (eg, subgroup and adjusted analyses)	______________
	20c	Definition of analysis population relating to protocol non-adherence (eg, as randomised analysis), and any statistical methods to handle missing data (eg, multiple imputation)	______________
**Methods: Monitoring**			
Data monitoring	21a	Composition of data monitoring committee (DMC); summary of its role and reporting structure; statement of whether it is independent from the sponsor and competing interests; and reference to where further details about its charter can be found, if not in the protocol. Alternatively, an explanation of why a DMC is not needed	__17___________
	21b	Description of any interim analyses and stopping guidelines, including who will have access to these interim results and make the final decision to terminate the trial	______________
Harms	22	Plans for collecting, assessing, reporting, and managing solicited and spontaneously reported adverse events and other unintended effects of trial interventions or trial conduct	_____17_________
Auditing	23	Frequency and procedures for auditing trial conduct, if any, and whether the process will be independent from investigators and the sponsor	______________
**Ethics and dissemination**			
Research ethics approval	24	Plans for seeking research ethics committee/institutional review board (REC/IRB) approval	___18__________
Protocol amendments	25	Plans for communicating important protocol modifications (eg, changes to eligibility criteria, outcomes, analyses) to relevant parties (eg, investigators, REC/IRBs, trial participants, trial registries, journals, regulators)	______________

Consent or assent	26a	Who will obtain informed consent or assent from potential trial participants or authorised surrogates, and how (see Item 32)	____18________
	26b	Additional consent provisions for collection and use of participant data and biological specimens in ancillary studies, if applicable	______________
Confidentiality	27	How personal information about potential and enrolled participants will be collected, shared, and maintained in order to protect confidentiality before, during, and after the trial	_____18_______
Declaration of interests	28	Financial and other competing interests for principal investigators for the overall trial and each study site	____21__________
Access to data	29	Statement of who will have access to the final trial dataset, and disclosure of contractual agreements that limit such access for investigators	____18__________
Ancillary and post-trial care	30	Provisions, if any, for ancillary and post-trial care, and for compensation to those who suffer harm from trial participation	______________
Dissemination policy	31a	Plans for investigators and sponsor to communicate trial results to participants, healthcare professionals, the public, and other relevant groups (eg, via publication, reporting in results databases, or other data sharing arrangements), including any publication restrictions	____17________
	31b	Authorship eligibility guidelines and any intended use of professional writers	______________
	31c	Plans, if any, for granting public access to the full protocol, participant-level dataset, and statistical code	______________
**Appendices**			
Informed consent materials	32	Model consent form and other related documentation given to participants and authorised surrogates	______________
Biological specimens	33	Plans for collection, laboratory evaluation, and storage of biological specimens for genetic or molecular analysis in the current trial and for future use in ancillary studies, if applicable	______________

*It is strongly recommended that this checklist be read in conjunction with the SPIRIT 2013 Explanation & Elaboration for important clarification on the items. Amendments to the protocol should be tracked and dated. The SPIRIT checklist is copyrighted by the SPIRIT Group under the Creative Commons "Attribution-NonCommercial-NoDerivs 3.0 Unported" license.

BMJ Open

Assessing the impact of a combined nutrition counselling and cash transfer intervention on women's empowerment in rural Bangladesh: A randomised control trial protocol.

Journal:	*BMJ Open*
Manuscript ID	bmjopen-2020-044263.R1
Article Type:	Protocol
Date Submitted by the Author:	22-Feb-2021
Complete List of Authors:	Kirkwood, Elizabeth; The University of Sydney Faculty of Medicine and Health, Sydney School of Public Health;<br>Dibley, Michael; The University of Sydney, School of Public Health<br>Hoddinott, John Frederick ; Cornell University,<br>Huda, Tanvir ; The University of Sydney, Sydney School of Public Health<br>Laba, Tracey; University of Technology Sydney, Centre for Health Economics Research and Evaluation<br>Tahsina, Tazeen; International Centre for Diarrhoeal Disease Research Bangladesh, Maternal and Child Health Division<br>Hasan, Mohammad; International Centre for Diarrhoeal Disease Research Bangladesh<br>Iqbal, Afrin; International Centre for Diarrhoeal Disease Research Bangladesh<br>Khan, Jasmin; International Centre for Diarrhoeal Disease Research Bangladesh<br>Ali, Nazia; International Centre for Diarrhoeal Disease Research Bangladesh<br>Ullah, Saad; International Centre for Diarrhoeal Disease Research Bangladesh<br>Goodwin, Nicholas; The University of Sydney<br>Muthayya, Sumithra; The Sax Institute<br>Islam, M; International Centre for Diarrhoeal Disease Research Bangladesh, Nutrition and Clinical Services Division<br>Ara, Gulshan; International Centre for Diarrhoeal Disease Research Bangladesh, NCSD<br>Agho, Kingsley ; Western Sydney University, School of Science and Health,<br>Arifeen, Shams E.; ICDDR B, Maternal and Child Health Division (MCHD)<br>Alam, Ashraful; University of Sydney, School of Public Health
**Primary Subject Heading**:	Public health
Secondary Subject Heading:	Global health
Keywords:	PUBLIC HEALTH, Protocols & guidelines < HEALTH SERVICES ADMINISTRATION & MANAGEMENT, Clinical trials < THERAPEUTICS

SCHOLARONE™
Manuscripts

I, the Submitting Author has the right to grant and does grant on behalf of all authors of the Work (as defined in the below author licence), an exclusive licence and/or a non-exclusive licence for contributions from authors who are: i) UK Crown employees; ii) where BMJ has agreed a CC-BY licence shall apply, and/or iii) in accordance with the terms applicable for US Federal Government officers or employees acting as part of their official duties; on a worldwide, perpetual, irrevocable, royalty-free basis to BMJ Publishing Group Ltd ("BMJ") its licensees and where the relevant Journal is co-owned by BMJ to the co-owners of the Journal, to publish the Work in this journal and any other BMJ products and to exploit all rights, as set out in our licence.

The Submitting Author accepts and understands that any supply made under these terms is made by BMJ to the Submitting Author unless you are acting as an employee on behalf of your employer or a postgraduate student of an affiliated institution which is paying any applicable article publishing charge ("APC") for Open Access articles. Where the Submitting Author wishes to make the Work available on an Open Access basis (and intends to pay the relevant APC), the terms of reuse of such Open Access shall be governed by a Creative Commons licence – details of these licences and which Creative Commons licence will apply to this Work are set out in our licence referred to above.

Other than as permitted in any relevant BMJ Author's Self Archiving Policies, I confirm this Work has not been accepted for publication elsewhere, is not being considered for publication elsewhere and does not duplicate material already published. I confirm all authors consent to publication of this Work and authorise the granting of this licence.

For peer review only

Author

Title

Assessing the impact of a combined nutrition counselling and cash transfer intervention on women's empowerment in rural Bangladesh: A randomised control trial protocol.

Authors

Elizabeth K. Kirkwood - ORCID 0000-0001-8603-4903

The University of Sydney, Sydney School of Public Health; Edward Ford Building, NSW, 2006, Australia.

elizabeth.kirkwood@sydney.edu.au

Michael J. Dibley - ORCID 0000-0002-1554-5180

The University of Sydney, Sydney School of Public Health; Edward Ford Building, NSW, 2006, Australia michael.dibley@sydney.edu.au

John Hoddinott – ORCID 0000-0002-0590-3917

Cornell University

Tanvir Huda – ORCID 0000-0002-8996-4361

The University of Sydney, Sydney School of Public Health; Edward Ford Building, NSW, 2006, Australia.

Tracey-Lea Laba – ORCID 0000-0002-5182-9092

University of Technology Sydney

Tazeen Tahsina – ORCID 0000-0001-5899-8422

International Centre for Diarrhoeal Disease Research, Bangladesh

Mohammad Mehedi Hasan – ORCID 0000-0003-0630-290X

International Centre for Diarrhoeal Disease Research, Bangladesh

Afrin Iqbal – ORCID 0000-0002-1859-8405

International Centre for Diarrhoeal Disease Research, Bangladesh

Jasmin Khan – ORCID 0000-0003-2022-6448

For peer review only - http://bmjopen.bmj.com/site/about/guidelines.xhtml

International Centre for Diarrhoeal Disease Research, Bangladesh

Nazia Binte Ali – ORCID 0000-0001-5010-9563

International Centre for Diarrhoeal Disease Research, Bangladesh

Saad Ullah - ORCID 0000-0001-7450-6483

International Centre for Diarrhoeal Disease Research, Bangladesh

Nicholas Goodwin – ORCID 0000-0003-2476-6310

The University of Sydney, Sydney School of Public Health; Edward Ford Building, NSW, 2006, Australia.

Sumithra Muthayya– ORCID 0000-0003-1645-9206

Sax Institute

Munirul Islam – ORCID 0000-0002-8780-8760

International Centre for Diarrhoeal Disease Research, Bangladesh

Gulshan Ara– ORCID 0000-0002-3745-5952

International Centre for Diarrhoeal Disease Research, Bangladesh

Kingsley Emwinyore Agho – ORCID 0000-0003-4111-3207

Western Sydney University

Shams El Arifeen – ORCID 0000-0002-5372-5932

International Centre for Diarrhoeal Disease Research, Bangladesh

Ashraful Alam - ORCID: 0000-0001-7034-1095

The University of Sydney, Sydney School of Public Health; Edward Ford Building, NSW, 2006, Australia neeloy.alam@sydney.edu.au

Corresponding author

Elizabeth K. Kirkwood

For peer review only - http://bmjopen.bmj.com/site/about/guidelines.xhtml

48 The University of Sydney, Sydney School of Public Health; Room 328 Edward Ford Building, NSW, 2006,
49 Australia. Phone: +61 410 517 874

50 Email: elizabeth.kirkwood@sydney.edu.au

For peer review only

For peer review only - http://bmjopen.bmj.com/site/about/guidelines.xhtml

Abstract

Introduction: There is growing interest in assessing the impact of health interventions, particularly when women are the focus of the intervention, on women's empowerment. Globally, research has shown that interventions targeting nutrition, health, and economic development can affect women's empowerment. Evidence suggests that women's empowerment is also an underlying determinant of nutrition outcomes. Depending on the focus of the intervention, different domains of women's empowerment will be influenced for example an increase in nutritional knowledge, or greater control over income and access to resources.

Objective: This study evaluates the impact of the Shonjibon Cash and Counselling trial that combines nutrition counselling and unconditional cash transfer intervention, delivered on a mobile platform, on women's empowerment in rural Bangladesh.

Methods and analysis: We will use a mixed-methods approach, combining statistical analysis of quantitative data from 2,080 women in a cluster randomised controlled trial examining the impact of nutrition behaviour change communications and cash transfers on child undernutrition. Pregnant participants will be given a smartphone with a customised app, delivering nutrition behaviour change communication messages, receive nutrition counselling via a call centre and an unconditional cash transfer. This study is a component of the Shonjibon Cash and Counselling trial and will measure women's empowerment using a composite indicator based on the Project-Level Women's Empowerment in Agriculture Index, with quantitative data collection at baseline and endline. Thematic analysis of qualitative data, collected through longitudinal interviews with women, husbands, and mothers-in-law, will elicit a local understanding of women's empowerment and the linkages between the intervention and women's empowerment outcomes. This paper describes the study protocol to evaluate women's empowerment in a nutrition-specific and sensitive-intervention using internationally validated, innovative

For peer review only - http://bmjopen.bmj.com/site/about/guidelines.xhtml

74 tools and will help fill the evidence gap on pathways of impact, highlighting areas to target for future
75 programming.

76 **Ethics and dissemination:**

77 Ethical approval has been obtained from the International Centre for Diarrhoeal Disease Research (Ref.
78 PR 17106) and The University of Sydney (Ref: 2019/840). Findings from this study will be shared in
79 Bangladesh with dissemination sessions in-country, internationally at conferences, and published in
80 peer-reviewed journals.

81 Word count: 5325

82 Keywords

83 Women's empowerment, nutrition, cash transfer, counselling, BCC, mHealth, protocol, Bangladesh.

Strengths and limitations of this study

- This paper describes the study protocol evaluating women's empowerment in a nutrition-specific and -sensitive RCT and will help fill the evidence gap on pathways of impact for women's empowerment and highlight areas to target for future policy and programming.
- Formative research guided this mixed-methods approach.
- Designed specifically tailored tools, based on a theory of change and using an internationally validated index, piloted in Bangladesh, indices that can provide global comparison.
- We are not using the Pro-WEAI in full as we interview women only for the quantitative component and will not calculate the Gender Parity Index, which index sheds light on the sense of intrahousehold inequality, however, we will explore this with qualitative research.

84

85 Background

86 To define women's empowerment is not straightforward, nor is it easy to measure across the different
87 domains and phases of a woman's life. Empowerment is "the process of enhancing an individual's or
88 group's capacity to make purposive choices and to transform those choices into desired actions and
89 outcomes"(1). The United Nations defines women's empowerment as having five key components:

For peer review only - http://bmjopen.bmj.com/site/about/guidelines.xhtml

"women's sense of self-worth; their right to have and to determine choices; their right to have access to opportunities and resources; their right to have the power to control their own lives, both within and outside the home; and their ability to influence the direction of social change to create a more just social and economic order, nationally and internationally" (2).

Gender, socially constructed roles assigned by sex, is one of the keynote determinants of health outcomes (3), making gender equality and the rights of women an imperative goal in global public health. Gender inequality and restrictive gender norms are, according to the Lancet 2019 series on Gender Equality, Norms and Health "powerful but separate determinants of health and well-being" (3). Sustainable Development Goal (SDG) 5 aims to achieve gender equality and empower all women and girls, ending discrimination in all forms; from violence to forced marriage, full active participation and equal rights in all spheres of life – politically, economically, and socially (4). Out of the 17 SDGs, 11 entail indicators related to gender.

Much progress has been made toward gender equality and the empowerment of women globally. Yet, the potential for women to fully participate as "agents of change" is still limited due to persistent social, economic and political inequalities (5). Women continue to be constrained by norms, beliefs, and customs through which societies differentiate between women and men (6). Contextual forms of oppression include patriarchal societies and institutions, as well as lack of opportunity due to socioeconomic circumstances such as poverty. Violence, including intimate partner violence (IPV), disproportionally affects women. Worldwide, a third of women report experience physical or sexual IPV (7). Women subjected to IPV suffer poor mental and physical health, and their children show poorer health, nutrition and development outcomes (8). Evidence suggests that the combination of cash transfers and behaviour change communication (BCC), can increase women's bargaining power and poverty-related emotional well-being and lead to a reduction in IPV (8).

For peer review only - http://bmjopen.bmj.com/site/about/guidelines.xhtml

Interventions to improve the health and nutrition literacy of women and, in particular, counselling through mobile phone applications can make a positive impact on women's empowerment. Mobile health (mHealth) interventions have the potential to improve a woman's confidence and decision-making skills in communicating with health care professionals. However, a systematic review revealed the need for a further rigorous investigation into mHealth, in terms of implementation and evaluation, to establish whether mHealth programs transform rather than reinforce gender inequalities (9). When a woman receives additional resources, such as cash transfers and is the target of a mHealth program this can challenge gender norms within relationships and exacerbate gender disparities (9). There is limited evidence on the adverse effects of mHealth, such as expanding the digital divide and gender-based power imbalances, and this lack of evidence emphasises the need to monitor the impact of mHealth interventions on gender relations and women's empowerment (9).

The Lancet Series on Maternal and Child nutrition also advocates the use of cash transfer and social safety net programmes, which are increasingly the preferred approach to support households living in chronic and extreme poverty (10). When targeted at women, social protection can promote women's economic empowerment and enhance decision-making ability, with the overarching assumption that control over cash will lead to greater investment in children's health and education (10, 11). When women are engaged in nutrition-sensitive programmes that utilise social safety nets, certain aspects of women's empowerment are augmented, such as changes in gender roles and intrahousehold bargaining power (10).

We plan to conduct a study, embedded in a cluster randomised control trial that assesses a multifaceted nutrition intervention on childhood stunting - Shonjibon Cash and Counselling Trial (SCC) (12). This protocol paper presents the way we will measure the impact of the SCC trial women's empowerment.

Developmental interventions and women's empowerment in Bangladesh

For peer review only - http://bmjopen.bmj.com/site/about/guidelines.xhtml

Although Bangladesh has made remarkable reductions in rates of poverty, almost 1 in 4 of the population still live in poverty (24.3% in 2016) (13). In 2018, the agriculture sector accounted for 40% of total employment (14). In most developing countries, women account for almost half of the agricultural labour force; however, in Bangladesh, women exceed fifty per cent of the agricultural labour force (15). In rural Bangladesh, social constructs such as gender roles of women and men powerfully drive household food consumption (16). A nationally representative sample from Bangladesh found that women's empowerment increases dietary diversity and availability of calories at a household level (17, 18). The Government of Bangladesh and development organisations have shown a keen interest in combining nutrition counselling and cash transfers to improve maternal and child nutrition (19).

In rural Bangladesh, 84% of households have access to a mobile phone (20). A study in Bangladesh revealed women using Aponjon, a mobile-messaging based service providing information about mother and babies, had higher rates of autonomy in accessing mobile phones, although two-thirds of the women resided in urban areas. mHealth consultations may help address socio-cultural gender norms, and empower women as they were able to discuss personal health issues on the phone with female doctors who are infrequently found in rural communities (20). The need to collect sex aggregated, valid, comprehensive and standardised empowerment data is therefore imperative to achieve not only SDG 5 but to enable the consistent measurement of women's empowerment indices (21, 22).

Objectives and Hypotheses

This study aims to evaluate the impact of a combined nutrition counselling and unconditional cash transfer intervention (Shonjibon Cash and Counselling / SCC Trial), delivered on a mobile platform, on the level of empowerment of women in rural Bangladesh. This study will assess women's experience of empowerment and disempowerment throughout the SCC trial.

For peer review only - http://bmjopen.bmj.com/site/about/guidelines.xhtml

The **primary hypothesis** is that in a community-based cluster randomised control trial of a mobile phone-based nutrition behaviour change communication (BCC) and unconditional cash transfer, given to women in low-income families in rural Bangladesh, women's empowerment will increase as measured by an increase in mean women's empowerment scores (by an average percentage of 20) from the baseline to the end of the 24-month intervention, compared to women in the control arm.

The **secondary hypotheses** are that the mobile phone nutrition BCC and unconditional cash transfer compared to usual programs will: a) increase control over income and economic resources; b) improve input and decision-making power in nutrition and health care choices; c) decrease the acceptance and experience of intimate partner violence.

SCC Trial women's empowerment Theory of Change

Empowering women is not an explicit goal of the SCC Trial; however, women gain access to nutrition counselling, cash transfers and a mobile phone; the utilisation of these resources can empower women. We constructed the theory of change to illustrate how the SCC Trial will impact on women's empowerment outcomes. It outlines the barriers, intervention details, intermediate outcomes, outcomes and impact pathways (Figure 1).

The SCC Trial intervention is delivered on a mobile platform. Women receive an mHealth interactive app – messages, audio and video, quizzes – as well as counselling from the call centre. They also receive cash via BKash mobile banking app. The principal rationale of the theory of change causal pathway to impact is that SCC Trial will lead to an increase in women's empowerment for those women participating in the intervention arm.

The Project-level Women's Empowerment in Agriculture Index (Pro-WEAI)(21) indicators, discussed in detail later, appear in the theory of change across the outcomes. We will use the six indicators from the Pro-WEAI to calculate the women's empowerment index and include: 1) Control over use of income; 2)

For peer review only - http://bmjopen.bmj.com/site/about/guidelines.xhtml

Input into productive decisions; 3) Autonomy in income; 4) Decision-making power on nutrition and health care; 5) Respect among household members; 6) Attitudes toward IPV.

One of the barriers to nutrition faced in rural Bangladesh is limited access to nutrition and infant and young child feeding (IYCF) information. Women in the SCC Trial intervention arm receive nutrition, IYCF and livelihood BCC delivered on a mobile app and nutrition counselling from the call centre. The intermediate outcome is woman's acquisition of new BCC/nutrition knowledge, contributing to increased input into and decision-making power about health and nutrition choices for herself and her family (Indicator 4). Improvements in communication also occur (spousal, intrahousehold, community) as new knowledge is discussed and shared, including ways to put the messages into action (Indicator 5). One's ability to make decisions is a measure of empowerment (23). New knowledge can be used as leverage and bargaining power and enhance decision-making abilities across a range of areas such as livelihoods, household expenditure and decisions on productive assets, i.e., what crops to farm (Indicator 2,3,4).

The SCC Trial setting is in one of the poorest regions in Bangladesh. Families face barriers such as food insecurity and lack of financial resources. When a woman is the recipient of a cash transfer, the intermediate outcome is increased control over the use of income (Indicator 1). Cash transfers, targeted at women, have the potential to improve livelihoods, alleviate poverty, enhance food security and increase women's empowerment (10). Spousal communication is also strengthened as discussion ensues as to how to spend the additional income (Indicator 4). The outcomes for women receiving an unconditional cash transfer include a strengthened ability to contribute to and participate in financial decision making. This decision-making covers health and nutrition choices, household expenditure, productive assets, livelihood choices (i.e. homestead gardening, buying chickens to raise for eggs, meat and income) (Indicator 1,2,3,4).

For peer review only - http://bmjopen.bmj.com/site/about/guidelines.xhtml

IPV includes behaviour that is physical, psychological, sexual or abusive or controlling in nature (24). There is evidence that the combination of additional income from cash transfers combined with BCC, can increase women's bargaining power and poverty-related emotional well-being (8). Changing attitudes, from within the family unit, as women participate and learn from intervention activities has the potential to lead to a change in the acceptability of IPV, linked to a woman's right to bodily integrity (25). Women may also experience an increase in respect and self-efficacy and subsequent reduction in acceptance of IPV (Indicator 6).

Figure 1 SCC Trial women's empowerment Theory of Change (adapted from De Silva et al. (26)

Study Design

We will embed this study on women's empowerment within the SCC Trial. The SCC Trial will recruit pregnant women and follow up over 24 months an expected 2,080 mother-child dyads, from recruitment in early pregnancy until the child is 18 months of age. The intervention will run for 36 months, depending on the time of enrolment and gestation, and age of the child.

Methods

This study will use a mixed-methods approach with data gathered on women's empowerment from participants in the SCC Trial. This protocol paper will adhere to the SPIRIT and Tidier guidelines for clinical trial protocols (27-29) to ensure rigour in protocol content.

Study setting

We will conduct the study in two subdistricts of Sirajganj; Ullapara and Kamarkhanda, in northern Bangladesh (Figure 2). Employment in Sirajganj is predominantly in agriculture (51 %) with 25 % working in services and 23% in industry (30).

Figure 2: Sirajganj, Bangladesh SCC Study site

For peer review only - http://bmjopen.bmj.com/site/about/guidelines.xhtml

For peer review only

Eligibility criteria

All married women between 15-49 years, that are permanent residents of the study area and provide consent to participate and test positive with pregnancy urine test kit (Excel®) (whose gestational age is ≤90 days) are eligible to participate in SCC Trial.

Intervention: Shonjibon[1] Cash and Counselling Trial

A separate protocol paper provides details of the design of the SCC Trial (12). Briefly, the SCC Trial aims to assess the effectiveness of nutrition behaviour change communication (delivered via call centre counselling and specifically tailored mobile app) combined with unconditional cash transfers in reducing the prevalence of stunting (length-for-age < -2 Z) in children at 24 months. We will use a longitudinal cluster randomised controlled trial to determine the effectiveness of unconditional cash transfers and mobile behaviour change communications (BCC) to reduce child undernutrition in Sirajganj district in northern Bangladesh, one of the poorest regions in the country according to the World Food Program Poverty Maps (31). Both quantitative and qualitative approaches will be employed to address the study objectives.

The SCC Trial intervention arm receives: 1) nutrition BCC delivered on a specially tailored app on a smartphone (audio, video, and animation), 2) direct nutrition counselling from a call centre and 3) unconditional cash transfer of 1000 Taka (USD 12.50) received monthly via BKash mobile banking app. The control arm will receive a mobile phone and the current Government of Bangladesh health and nutrition services. The participants in the control arm receive a mobile phone as the trial is interested in the effects of the messages, nutrition counselling and cash transfer delivered on a mobile platform, as opposed to the non-specific effects of a mobile phone.

[1] [1]Shonjibon stands for Shustho Notun Jibon in Bangla, translated, it means "Healthy New Life"

For peer review only - http://bmjopen.bmj.com/site/about/guidelines.xhtml

Study outcomes

The primary study outcome will be the difference in women's empowerment scores in the intervention arm as compared to the control arm of the SCC Trial. Secondary study outcomes will include 1) control over income and economic resources; 2) input and decision-making power in nutrition and health care choices; 3) experience and attitude toward intimate partner violence.

Outcome measurements

The SCC trial will collect data across the main module of the Pro-WEAI (12 indicators) as well as the additional health and Pro-WEAI Nutrition and Health module. However, 6 of the Pro-WEAI indicators are likely to be directly impacted by the SCC trial, therefore, we will develop a composite women's empowerment indicator based on the following: 1) control over the use of income; 2) input in production decisions; 3) autonomy in income; 4) decision making power about women and children's health and nutrition; 5) respect among household members and 6) attitudes toward IPV (Appendix Table 1). We will also measure women's and family member's perceived and lived experiences related to women empowerment qualitatively (Appendix Table 1).

1. Control over the use of income

We will measure control over the use of income by asking questions from Pro-WEAI; these questions are significant as the SCC Trial gives an unconditional cash transfer to the woman. Quantitative questions assess a woman's input into decision making on all sources of household income. Qualitative interviews will explore local perceptions of intrahousehold harmony and communication skills, issues related to withdrawing the cash, and perceptions and experience of control and expenditure of the money.

2. Input in productive decisions

For peer review only - http://bmjopen.bmj.com/site/about/guidelines.xhtml

These Pro-WEAI questions centre around input into household decision making on areas such as livelihood choices (activities the household participate in such as agricultural activities, home gardens etc), employment and household purchases.

3. Autonomy in income

A series of vignettes, validated by cognitive testing, using a series of hypothetical scenarios to assess motivation as to how income is used.

4. Decision-making ability about health and nutrition choices

We will examine responses from the Pro-WEAI Nutrition and Health Module regarding confidence and participation in decisions about nutrition and infant feeding, as well as a range of issues affecting women and children such as whether to consult a doctor when ill to dietary choices when pregnant.

5. Respect among household members

Questions in this indicator pertain to a woman's spouse or other household members on respect and trust within relationships, and level of comfort in disagreeing with household members.

6. Intimate Partner Violence

Previous research in Bangladesh has found that cash transfers and behaviour change communication can reduce IPV (8). We will measure a woman's attitude toward IPV using the Pro-WEAI as well as the direct experience of IPV using the questions from the Violence Against Woman and Girls 2015 Survey (32, 33). We will also conduct in-depth interviews to explore the woman's attitudes and perceptions of IPV qualitatively.

Participant recruitment and timeline

For peer review only - http://bmjopen.bmj.com/site/about/guidelines.xhtml

A household census in the study area will record the names and contact details of all women that agree to participate, and we will enter their details into an electronic system. The system will generate for each participant with a unique ID number and quick response (QR) code. SCC surveillance workers will then conduct door-to-door visits each month to identify women that have missed two menstrual periods in a row. The woman will then undertake a pregnancy test (using a urine test kit (Excel®). We will invite women who test positive for pregnancy to participate in the study with appropriate informed written consent. Based on experience with our pilot study, we anticipate 95% of the mothers will consent. Qualitative and quantitative data will be gathered at the start of the intervention and at endline (Appendix Table 2).

Allocation of clusters

Our study site has over 1100 villages, which would be adequate to cover the anticipated sample size. Each cluster encompasses 2-4 villages and consists of ~1000 households and ~4500 population. We will use a fixed randomization scheme to assign treatments to eligible clusters, with a uniform allocation ratio of treatments, stratified by Union. The sample size will consist of 104 clusters, total women (N = 2080) allocated to two parallel groups (n = 1040) with 20 mother-infant dyads per cluster. Each cluster consists of 1-4 adjacent villages with a minimum population of 600 households per cluster with 454 villages in our study area for clustering. A subsample of the households from a purposively selected subset of clusters will be chosen for qualitative data.

The intervention and control groups will receive similar maternal and child health and nutrition services from the government, and the critical difference will be the nutrition behaviour change communications with cash transfers on a mobile platform received by women in the intervention arm. The study design will control for potential observed and unobserved confounding factors as there will be an adequate number of clusters randomly allocated to the treatment groups. The geographic separation of clusters will

For peer review only - http://bmjopen.bmj.com/site/about/guidelines.xhtml

limit the contamination of the intervention arm. We will use Stata® software to generate the random allocation sequence. We are unable to mask the treatment arm due to the nature of the intervention.

Sample Size and power

The sample size for our trial is fixed by the primary hypothesis in the main Shonjibon Cash and Counselling trial, which estimated a total sample of 2184 mother-infant pairs from 104 clusters (12). We can find no reports of intra cluster correlation coefficients (icc) for the women's empowerment indicators we plan to use. Therefore, we estimate that the fixed trial sample size will provide at least 80% power to detect a 20% increase in women's empowerment assuming 30% of women are empowered (ref) and a high icc of 0.2. Assuming a lower icc of 0.05 we will have 90% power to detect a 13% increase in women's empowerment.

Data Collection methods

The SCC Trial will collect key household data and nutrition indices, while this study collects data on women's empowerment using the Pro-WEAI, whose focus is measuring agency for which there are limited if any standardised measures (21). We will include the Pro-WEAI nutrition and health module and supplementary questions regarding intimate partner violence. The qualitative component will be tailored to context-specific issues and guided by formative research.

Quantitative data

We will collect the women's empowerment indicators in household surveys at the baseline and the endline of the 24-month study in the intervention and non-intervention arms. We will pre-test all questions before the commencement of the study. We will use the validated short version of Marlowe-Crowne's social desirability scale (34), which is based on a subset of 13 items from the original scale. We will calculate a social desirability score by adding up the number of socially desirable answers, out of the

For peer review only - http://bmjopen.bmj.com/site/about/guidelines.xhtml

331 13 questions. The potential range of the score will be from 0-13 and we will create three categories with a score of 0-4 graded as a low score, 5-9 as a medium score, and 10-14 as a high score (Appendix Table 3). The questionnaire assesses whether social desirability bias or the tendency of respondents to answer questions in a way viewed favourably by the research team has influenced the data collected. We will train the field researchers in detecting social desirability bias in qualitative data and limit bias by properly introducing the study to the respondents, building rapport and asking context-specific probing questions following the recent framework described by Bergen and Labonté (35). We will follow the process throughout the research.

Qualitative data

The study uses qualitative analysis to elicit a local understanding of women's empowerment and the linkages between the SCC Trial and women's empowerment outcomes. We will generate qualitative data on women's and men's perceived and experienced change in empowerment through longitudinal qualitative semi-structured in-depth interviews. We will also interview key family members as suggested by the women, for example, husband or mother-in-law.

We will explore the myriad of ways that women and men perceive and describe empowerment in their communities. A purposive sample will be interviewed approximately two months after the start of the intervention and at the endline of the SCC Trial. Due to the highly sensitive nature of the topics, a separate team of interviewers, from a different Upazila, will be trained to collect data on women's empowerment and intimate partner violence. We will train the team in line with the ethical and safety procedures as per the World Health Organization's guidelines (36). The training will cover measures such as changing topics and asking a decoy nutrition question if anyone comes into the interview space and having details of referral and support services for women affected by or experiencing IPV.

Pro-WEAI

For peer review only - http://bmjopen.bmj.com/site/about/guidelines.xhtml

354 The Pro-WEAI measures the empowerment of women, agency and inclusion in the agriculture sector (21).
355 It is a standardised, survey-based internationally validated index, developed to adapt to project-specific
356 contexts and has been piloted in Bangladesh (17, 21). This study uses Kabeer's conceptual framework of
357 empowerment focuses on the ability of individuals to make choices, ''a process of change during which
358 those who have been denied the ability to make choices acquire such an ability'' (37, 38). Agency or the
359 ability of the individual to make considered choices is at the centre of the framework and revolves around
360 interrelated dimensions of resources, agency and achievements. This framework underpins the Pro-WEAI
361 (21). This study will collect data from women, across twelve indicators that are equally weighted (Figure
362 3), with the addition of the Project-specific Nutrition and Health module.

363 *Figure 3: Pro-WEAI 3 Domains of Empowerment and 12 Indicators (21)*

364 *Intimate Partner Violence*

365 The Pro-WEAI includes questions on a woman's attitude to intimate partner violence (IPV). We will collect
366 additional data about personal experience and behaviour specific questions regarding IPV, as opposed to
367 her perceptions thereof. We took these questions from the WHO Violence Against Women and Girls
368 Survey (Bangladesh 2015) (33). We will also ask questions relating to cyberviolence (39).

369 *Health and Nutrition*

370 The Pro-WEAI offers the option of adding project-specific modules, and for this study, we will add the
371 Nutrition and Health Module. This module asks questions about decision making about nutrition and
372 health indices for women and children.

373 Data collection and management

374 Intervention and evaluation staff will be separate to and not know the trial hypothesis to minimise
375 assessment bias. Android Tablets will be used in the field to enable interactive data collection. During each

For peer review only - http://bmjopen.bmj.com/site/about/guidelines.xhtml

evaluation interview, the data collectors will be guided and navigated by the software. As soon as the field staff complete each interview, they will upload the data to the server at icddr,b. The data will be retained on the tablet and copied/merged with files at icddr,b., with android tabs being synchronised with the server daily. We will systematically scrutinise the captured data and address any discrepancies as they occur. Qualitative narrative data will be captured in digital audio recorders and stored in the server from where we will check for quality and improvement for future interviews.

Quantitative Data Analysis

Data analysis will be by intention to treat. The women's empowerment composite index scores will be categorized as the percentage of women as empowered or disempowered. For each empowerment indicator individuals are classified as adequate or inadequate based on Pro-WEAI predetermined thresholds. Women's are considered empowered if 4 out of 6 indicators are adequate. We will also analyze impact of SCC intervention on individual indicators to assess increases or decreases in empowerment as not all indicators will respond or be impacted in the same way.

Analyses will be conducted at the mother-infant dyad level but will be adjusted for the cluster randomization (40). Primary analyses will compare the prevalence of women's empowerment at the end of the trial using Pearson's chi-square tests and 95% confidence intervals for the group difference, adjusted for clustering and generalized linear mixed models for non-continuous outcomes (e.g. logistic mixed models for binary outcomes e.g. percentage of women's empowerment). Models will include treatment group as a fixed effect, infants as a random effect to account for repeated measurements, and community-cluster as a random effect to account for cluster effects. We will also assess if the women's empowerment level at baseline, age, education and presence or absence of mother-in-law in the household modify the empowerment response to the intervention by testing for interactions between the intervention and these factors. STATA® will be used for all analyses.

For peer review only - http://bmjopen.bmj.com/site/about/guidelines.xhtml

399 **Qualitative Data Analysis**

400 We will record all interviews using digital audio recording devices. Interviews will be transcribed verbatim in Bangla by the research team at icddr,b. We will maintain quality control, by checking the transcripts at random intervals, to ensure the quality and accuracy of transcriptions. Data will be coded using thematic analysis approach (41). We will use NVivo software to organise, code, categorise and compile the data. Qualitative methods offer an effective way of describing in-depth, complex and varied perceptions of empowerment within the local context. They will also aid with the interpretation of individual indicators within the quantitative data. Data triangulation by using different sources of data will add strength to the analysis (42).

408 **Data safety and monitoring board**

409 An independent data and safety monitoring board (DSMB) will be formed to assess the completeness and quality of data and to ensure data is compliant with recruitment and retention goals. The DSMB will also assess any factors that might affect the study outcome or compromise the confidentiality of the trial data. Any unintended effects of the trial will be reported to the board.

413 **Patient and Public Involvement**

414 The development of the research question and outcome measures was informed by patients' priorities, experience, and preferences based on our formative research and pilot study, whereby we engaged the local community and sought their views about the proposed intervention. Patients were involved in the design of the study by taking part in the formative research. Patients were not involved in the recruitment and conduct of the study. The key trial findings will be disseminated to study participants through meetings with community gatekeepers and local administration. In this RCT, the burden of the intervention assessed by patients themselves as we undertook a pilot study.

For peer review only - http://bmjopen.bmj.com/site/about/guidelines.xhtml

421 **Dissemination plan**

422 Lessons learned from this study will be shared in Bangladesh with dissemination sessions in-country.
423 Internationally, key findings will be shared with stakeholders, results will be presented at conferences,
424 and published in international peer-reviewed journals.

425 **Access to data**

426 The SCC investigators will have access to all data and the right to analyse and publish data. Datasets will
427 be shared after all personally identifiable information has been removed and to keep the identification of
428 study subjects in confidence.

429 **Ethics approval and consent**

430 We have obtained approval for the SCC Trial from the Ethical Review Committee (ERC) of the International
431 Centre for Diarrhoeal Disease Research (icddr,b) – (Ref. PR 17106), and the Human Research Ethics
432 Committees (HRECs) at The University of Sydney (Ref: 2019/840).

433 We will train the women on the use of smartphones as well as the nutrition counselling application. We
434 will store the data obtained on the use of the application with access to limited authorised project staff.
435 Trained field staff will educate the women about the safety procedures for receipt and use of money. We
436 will record responses anonymously and by identification number. We will maintain the confidentiality,
437 privacy, and anonymity of the participants.

438 Project field workers will explain the nature, purpose, benefits, risks, and process of the trial to potential
439 participants as they register themselves in the surveillance system if they meet the inclusion criteria. We
440 will train them to obtain written informed consent (in Bangla) from all intervention participants.
441 Participation in the research will be voluntary, and the respondent will have every right to withdraw

For peer review only - http://bmjopen.bmj.com/site/about/guidelines.xhtml

anytime throughout the programme without any obligation, loss or penalty. We will explain the participant's rights before collecting any data.

Discussion

The main goal of this study is to evaluate the impact of a combined nutrition counselling and unconditional cash transfer intervention, delivered on a mobile platform, on women's empowerment in rural Bangladesh. We recognise that women's empowerment is an essential goal in global public health and the key to sustainable development. It is therefore crucial, that in complex and large-scale nutrition interventions, such as the SCC Trial, that we measure pathways of impact across the domains of women's empowerment. The 2013 Lancet Series on Maternal and Child Nutrition proposed utilising interventions that raise women's overall level of empowerment (10). Research has shown evidence of the impact of various development interventions on women's empowerment (43-45). A 2018 analysis of surveys from 54 countries observed that in critical aspects of family relationships, four out of five women did not hold agency (46). Cunningham et al. (2015) (47) note that women lack not only the resources to make significant decisions leading to better health and nutritional outcomes, but when disempowered, they also lack the autonomy and decision-making power within the household to make these critical decisions, having a multiplier effect on family and community.

This study has several strengths; firstly, it is embedded within a cluster RCT that aims to assess the effectiveness of a nutrition behaviour change communication combined with unconditional cash transfers in reducing the prevalence of stunting. Another strength lies in the use of a mixed-methods approach – obtaining qualitative and quantitative data. We gain further advantage from the use of tools tailored specifically for this intervention; tools that are based on formative research, wide-ranging literature review and piloted in Bangladesh, providing accurate, comprehensive and appropriate methodology.

For peer review only - http://bmjopen.bmj.com/site/about/guidelines.xhtml

The Pro-WEAI is designed to assess the impact evaluation of agricultural development projects (48). The SCC Trial is not an agricultural intervention, however, women in Bangladesh characteristically do postharvest activities and processing yet do not classify themselves as agricultural workers. It is in this context that we are using the index, to survey women in Bangladesh, whose livelihoods are bound to the agricultural sector (49). Alkire (2018) describes the Pro-WEAI as an "information platform" that is applicable in broader contexts other than exclusively agricultural interventions (50). The core elements of the SCC women's empowerment index, a customised tool, will provide translatable results – using measures and indices that allow global comparisons.

When the Pro-WEAI is used in full – women and men are interviewed - this enables the calculation of the Gender Parity Index, which measures empowerment and disempowerment within the household. The limitation of not interviewing men is that we may not capture the sense of intrahousehold inequality - are the women at least as empowered as the men in their households? We will account for this by ensuring that qualitative exploration delves into the extent to which the disempowerment observed is attributable to gender norms versus other causes. The SCC Trial does not directly focus on women's empowerment nor contest existing gender norms or patriarchal power structures. The study participants - the recipients of the cash transfer, nutrition counselling and smartphone - are, however, women. This study will identify where gaps in empowerment occur, highlight key areas of disempowerment and which domains help facilitate or hinder nutrition outcomes.

Funding

The Shonjibon Cash and Counselling Trial is funded by the National Health and Medical Research Council of Australia GNT 1120507. EKK received seed funding for a field visit from the University of Sydney Faculty of Medicine and Health. The funders do not have any role in the study design, data collection, and interpretation of data.

For peer review only

Trial registration:

- The Shonjibon Cash and Counselling Trial is registered with the Australian New Zealand Clinical Trials Registry (ANZCTR) - The registration ID is ACTRN 12618001975280.

Abbreviations

- BCC behaviour change communication
- DSMB Data Safety Monitoring Board
- FGD focus group discussions
- icddr,b International Centre for Diarrhoeal Disease Research
- IDI in-depth-interview (IDI)
- IFPRI International Food Policy Research Institute
- IPV intimate partner violence
- IYCF infant young child feeding
- NHMRC National Health and Medical Research Council of Australia
- Pro-WEAI Project-Level Women's Empowerment in Agriculture Index
- SCC Trial Shonjibon Cash and Counselling Trial

For peer review only

Author's contributions

Elizabeth K Kirkwood drafted the paper, designed the study tools and main conceptual ideas. Ashraful Alam and Michael Dibley made continuous contributions and supervised in the design of the study and throughout the writing. All other authors (JH, TH, TLL, TT, MMH, AI, JK, NBA, SA, NG, SM, MI, GA, KEA, and SA were involved in developing the main study design and methods and read, critically revised, and approved the final manuscript, and met the ICMJE criteria for authorship.

Acknowledgements

For peer review only - http://bmjopen.bmj.com/site/about/guidelines.xhtml

509 We gratefully acknowledge the team at the International Food Policy Research Institute for their
510 assistance and sharing the Project-Level Women's Empowerment in Agriculture Index. I would also like to
511 thank the seminar attendees at the Sydney School of Public Health's Global Health and Nutrition Research
512 Collaboration for providing valuable feedback at my presentation on the tools and methodology used in
513 this paper.

514 **Competing Interests**

515 The authors declare that they do not have any competing interests.

516

517

For peer review only

For peer review only - http://bmjopen.bmj.com/site/about/guidelines.xhtml

References

1. Alsop R, Bertelsen MF, Holland J. Empowerment in practice - from analysis to implementation Washington, DC 20433 USA; 2006.

2. Department of Economic and Social Affairs UPD. Guidelines on Women's Empowerment. New York, USA: United Nations; 2010.

3. Heise L, Greene ME, Opper N, Stavropoulou M, Harper C, Nascimento M, et al. Gender inequality and restrictive gender norms: framing the challenges to health. The Lancet. 2019;393(10189):2440-54.

4. Transforming Our World: The 2030 Agenda for Sustainable Development. . United Nations, New York, USA.: United Nations; 2015. Contract No.: 09/10/2018.

5. The Future We Want. Rio + 20, Rio de Janeiro, Brazil: United Nations; 2012.

6. Kabeer N. The Power to Choose: Bangladeshi Women and Labour Market Decisions in London and Dhaka: Verso Books; 2000.

7. Global and regional estimates of violence against women: prevalence and health effects of intimate partner violence and non-partner sexual violence. Geneva: World Health Organization; 2013.

8. Roy S, Hidrobo M, Hoddinott J, Ahmed A. Transfers, Behavior Change Communication, and Intimate Partner Violence: Postprogram Evidence from Rural Bangladesh. The Review of Economics and Statistics. 2019;101(5):865-77.

9. Jennings L, Gagliardi L. Influence of mhealth interventions on gender relations in developing countries: a systematic literature review. International Journal for Equity in Health. 2013;12(1):85.

10. Ruel MT, Alderman H. Nutrition-sensitive interventions and programmes: how can they help to accelerate progress in improving maternal and child nutrition? The Lancet. 2013;382(9891):536-51.

11. Campos APdlO. Empowering rural women through social protection. Rome: FAO - Food and Agriculture Organization of the United Nations

2015.

12. Huda TM, Alam A, Tahsina T, Hasan MM, Iqbal A, Khan J, et al. Shonjibon cash and counselling: a community-based cluster randomised controlled trial to measure the effectiveness of unconditional cash transfers and mobile behaviour change communications to reduce child undernutrition in rural Bangladesh. BMC Public Health. 2020;20(1):1776.

13. Headey D, Hoddinott J, Ali D, Tesfaye R, Dereje M. The Other Asian Enigma: Explaining the Rapid Reduction of Undernutrition in Bangladesh. World Development. 2015;66:749-61.

14. Employment in agriculture (% of total employment) (modeled ILO estimate) [Internet]. 2018 [cited 13/05/2019]. Available from: https://data.worldbank.org/indicator/SL.AGR.EMPL.ZS?locations=BD.

15. Women Farmers of Bangladesh. Food and Agriculture organization of the United Nations; 2016.

16. Patalagsa MA, Schreinemachers P, Begum S, Begum S. Sowing seeds of empowerment: effect of women's home garden training in Bangladesh. Agriculture & Food Security. 2015;4(1):24.

17. Alkire S, Meinzen-Dick R, Peterman A, Quisumbing A, Seymour G, Vaz A. The Women's Empowerment in Agriculture Index. World Development. 2013;52:71-91.

18. Sraboni E, Malapit HJ, Quisumbing AR, Ahmed AU. Women's Empowerment in Agriculture: What Role for Food Security in Bangladesh? World Development. 2014;61:11-52.

19. Huda TM, Alam A, Tahsina T, Hasan MM, Khan J, Rahman MM, et al. Mobile-Based Nutrition Counseling and Unconditional Cash Transfers for Improving Maternal and Child Nutrition in Bangladesh: Pilot Study. JMIR mHealth and uHealth. 2018;6(7):e156-e.

For peer review only

For peer review only - http://bmjopen.bmj.com/site/about/guidelines.xhtml

20. Alam M, Banwell C, Olsen A, Lokuge K. Patients' and Doctors' Perceptions of a Mobile Phone–Based Consultation Service for Maternal, Neonatal, and Infant Health Care in Bangladesh: A Mixed-Methods Study. JMIR Mhealth Uhealth. 2019;7(4):e11842.
21. Malapit HJ, Quisumbing AR, Meinzen-Dick RS, Seymour G, Martinez EM, Heckert J, et al. Development of the Project-Level Women's Empowerment in Agriculture Index (Pro-WEAI). IFPRI; 2019 January 18, 2019.
22. Weber AM, Cislaghi B, Meausoone V, Abdalla S, Mejía-Guevara I, Loftus P, et al. Gender norms and health: insights from global survey data. The Lancet. 2019;393(10189):2455-68.
23. Seymour G, Peterman A. Context and measurement: An analysis of the relationship between intrahousehold decision making and autonomy. World Development. 2018;111:97-112.
24. Violence against women. World Health Organization; 2017.
25. Malapit H, Quisumbing A, Meinzen-Dick R, Seymour G, Martinez EM, Heckert J, et al. Development of the project-level Women's Empowerment in Agriculture Index (pro-WEAI). World Development. 2019;122:675-92.
26. De Silva MJ, Breuer E, Lee L, Asher L, Chowdhary N, Lund C, et al. Theory of Change: a theory-driven approach to enhance the Medical Research Council's framework for complex interventions. Trials. 2014;15(1):267.
27. Hoffmann Tammy C GPP, Boutron Isabelle, Milne Ruairidh, Perera Rafael, Moher David, Virginia Barbour, Marie Johnston, Sarah E. Lamb, Mary Dixon-Woods, Jeremy C. Wyatt. Better reporting of interventions: template for intervention description and replication (TIDieR) checklist and guide. BMJ. 2014:348:g1687.
28. Chan A-W, Tetzlaff JM, Altman DG, Laupacis A, Gøtzsche PC, Krleža-Jerić K, et al. SPIRIT 2013 Statement: Defining Standard Protocol Items for Clinical Trials. Annals of Internal Medicine. 2013;158(3):200-7.
29. Chan A-W, Tetzlaff JM, Gøtzsche PC, Altman DG, Mann H, Berlin JA, et al. SPIRIT 2013 explanation and elaboration: guidance for protocols of clinical trials. BMJ : British Medical Journal. 2013;346:e7586.
30. Bangladesh Interactive Poverty Maps: The World Bank; 2016 [Available from: http://www.worldbank.org/en/data/interactive/2016/11/10/bangladesh-poverty-maps.
31. Poverty Maps of Bangladesh 2010. World Food Programme (WFP); 2010 2010.
32. García-Moreno C, Jansen HAFM, Ellsberg M, Heise L, Watts C. WHO multi-country study on women's health and domestic violence against women. Geneva: World Health Organization; 2005.
33. Report on Violence Against Women Survey 2015. Dhaka, Bangladesh: Bangladesh Bureau of Statistics, Statistics and Informatics Division, Ministry of Planning, Government of the People's Republic of Bangladesh.
34. Reynolds WM. Development of reliable and valid short forms of the marlowe-crowne social desirability scale. Journal of Clinical Psychology. 1982;38(1):119-25.
35. Bergen N, Labonté R. "Everything Is Perfect, and We Have No Problems": Detecting and Limiting Social Desirability Bias in Qualitative Research. Qualitative Health Research. 2020;30(5):783-92.
36. Ellsberg M, Heise L. Researching Violence Against Women: A Practical Guide for Researchers and Activists. Washington DC, United States: World Health Organization, PATH; 2005.
37. Kabeer N. Resources, Agency, Achievements: Reflections on the measurement of Women's Empowerment. Development and Change. 1999;30(3):435-64.
38. Kabeer N. Gender equality and women's empowerment: A critical analysis of the third millennium development goal 1. Gender & Development. 2005;13(1):13-24.
39. Navid RT, Samuels F, Le MAsson V, Talukder A, Gupta T, Yount KM. Understanding intimate partner violence in rural Bangladesh: Prevention and response. London: ODI, Emory University, icddr,b; 2017 May 2017.

For peer review only - http://bmjopen.bmj.com/site/about/guidelines.xhtml

40. Hayes R, Moulton L. Cluster Randomised Trials. New York, USA: Taylor & Francis; 2009.

41. Braun V, Clarke V. Using thematic analysis in psychology. Qualitative Research in Psychology. 2006;3(2):77-101.

42. Guion L, Diehl D, McDonald D. Triangulation: Establishing the Validity of Qualitative Studies. University of Florida, The Institute of Food and Agricultural Sciences (IFAS) 2011.

43. Kim JC, Watts CH, Hargreaves JR, Ndhlovu LX, Phetla G, Morison LA, et al. Understanding the impact of a microfinance-based intervention on women's empowerment and the reduction of intimate partner violence in South Africa. Am J Public Health. 2007;97(10):1794-802.

44. Kabeer N. Women's Empowerment, Development Interventions and the Management of Information Flows. Institute of Development Studies. 2010;41(6).

45. Malhotra A, Schuler SR. Women's Empowerment as a Variable in International Development. In: Bank TW, editor. Measuring Empowerment: Cross Disciplinary Perspectives2005. p. 71- 88.

46. Heymann J, Levy JK, Bose B, Ríos-Salas V, Mekonen Y, Swaminathan H, et al. Improving health with programmatic, legal, and policy approaches to reduce gender inequality and change restrictive gender norms. The Lancet. 2019;393(10190):2522-34.

47. Cunningham K, Ruel MT, Ferguson E, Uauy R. Women's empowerment and child nutritional status in South Asia: a synthesis of the literature. Maternal & Child Nutrition. 2015;11(1):1-19.

48. Yount KM, Cheong YF, Maxwell L, Heckert J, Martinez EM, Seymour G. Measurement Properties of the Project-Level Women's Empowerment in Agriculture Index. International Food Policy Research Institute; 2019.

49. WEAI FAQs: International Food Policy Research Institute 2018 [Available from: https://weai.ifpri.info/weai-resource-center/faqs-2/.

50. Alkire S. Multidimensional Poverty Measures as Relevant Policy Tools. Oxford Poverty & Human Development Initiative 2018.

For peer review only - http://bmjopen.bmj.com/site/about/guidelines.xhtml

636

For peer review only

For peer review only - http://bmjopen.bmj.com/site/about/guidelines.xhtml

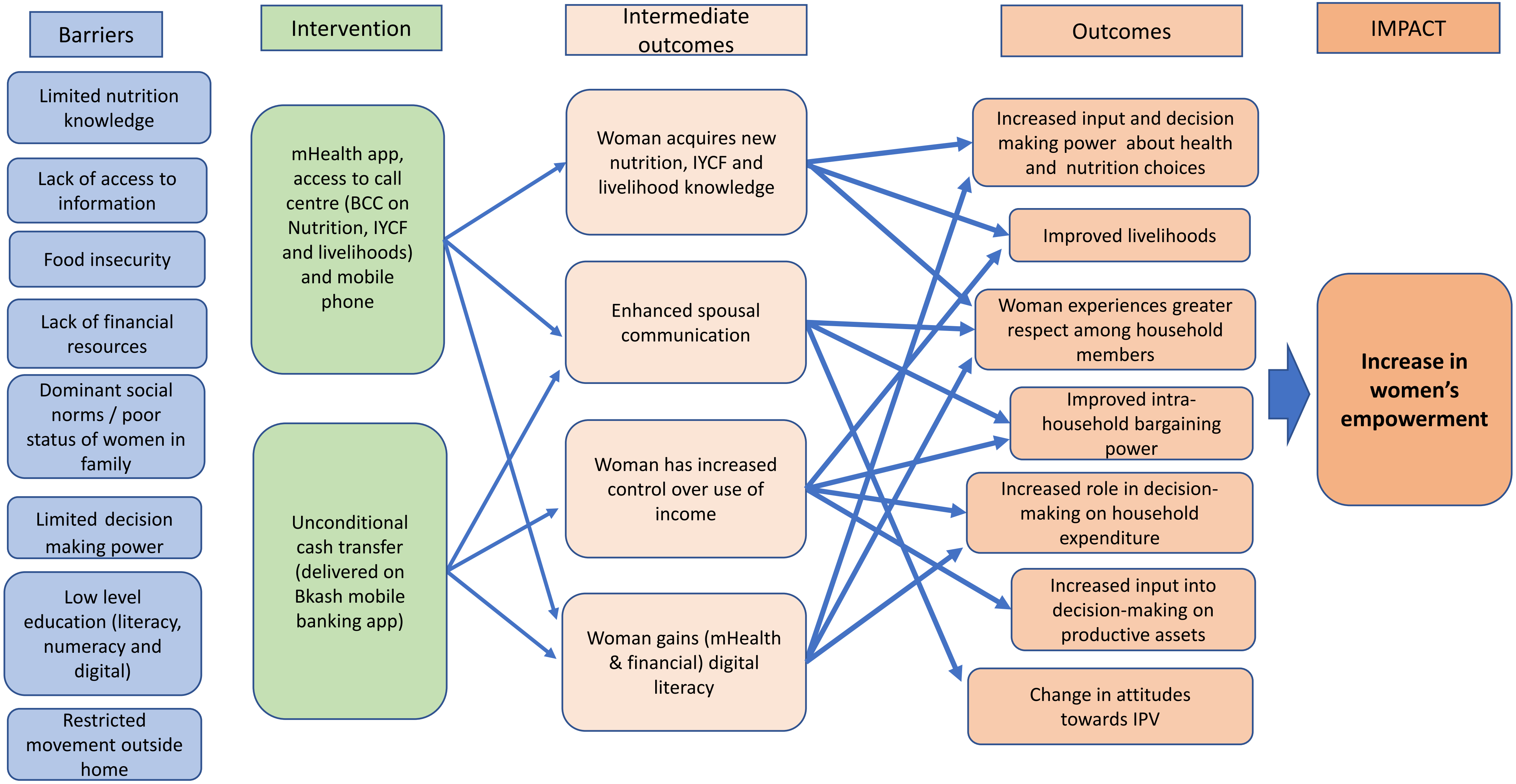
Barriers
Limited nutrition knowledge
Lack of access to information
Food insecurity
Lack of financial resources
Dominant social norms / poor status of women in family
Limited decision making power
Low level education (literacy, numeracy and digital)
Restricted movement outside home
Intervention
mHealth app, access to call centre (BCC on Nutrition, IYCF and livelihoods) and mobile phone
Unconditional cash transfer (delivered on Bkash mobile banking app)
Intermediate outcomes
Woman acquires new nutrition, IYCF and livelihood knowledge
Enhanced spousal communication
Woman has increased control over use of income
Woman gains (mHealth & financial) digital literacy
Outcomes
Increased input and decision making power about health and nutrition choices
Improved livelihoods
Woman experiences greater respect among household members
Improved intra-household bargaining power
Increased role in decision-making on household expenditure
Increased input into decision-making on productive assets
Change in attitudes towards IPV
IMPACT
Increase in women's empowerment

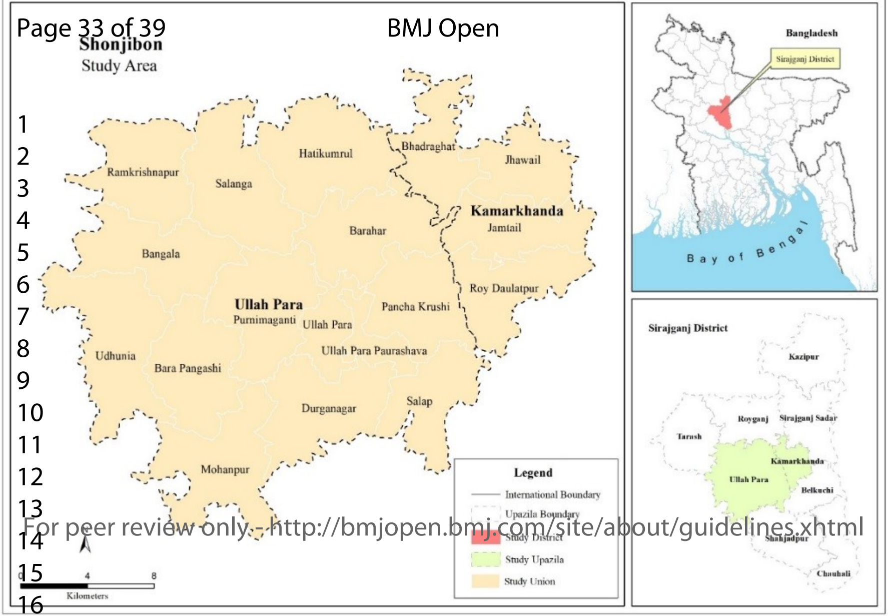

For peer review only - http://bmjopen.bmj.com/site/about/guidelines.xhtml

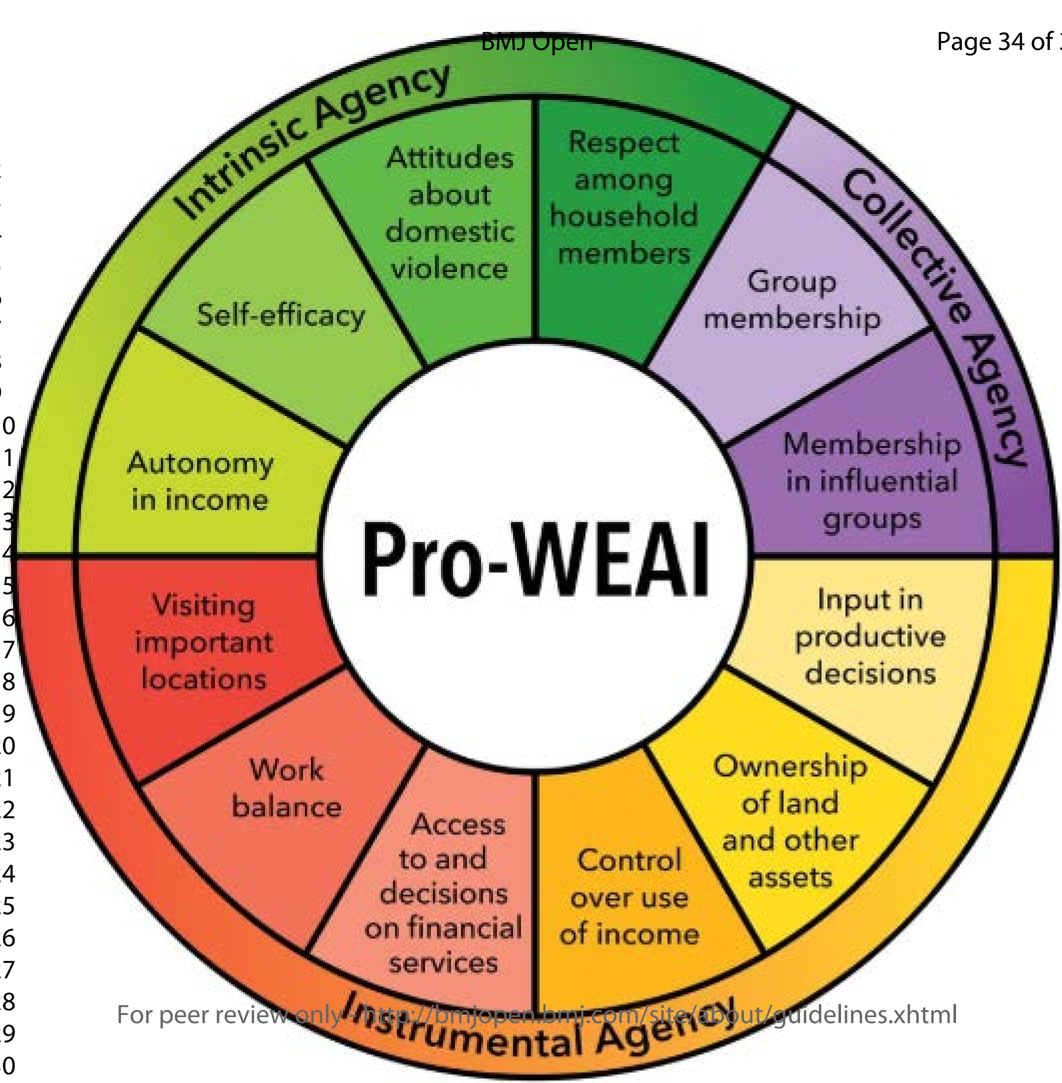
Intrinsic Agency
Attitudes about domestic violence
Respect among household members
Collective Agency
Self-efficacy
Group membership
Autonomy in income
Membership in influential groups
Pro-WEAI
Visiting important locations
Input in productive decisions
Work balance
Ownership of land and other assets
Access to and decisions on financial services
Control over use of income
Instrumental Agency

Appendix

Table 1: SCC women's empowerment summary index components

Shonjibon Trial resources	Pathway	Outcome variable / index components
mHealth app, access to call centre (BCC on Nutrition, IYCF and livelihoods) and mobile phone<br><br>Unconditional cash transfer (delivered on Bkash mobile banking app)	Instrumental Agency (Power to)	1. Control over use of income (G2)<br>2. Input in production decisions (G2)
	Intrinsic agency (power within)	3. Autonomy in income (G8(a))<br>4. Decision-making power on nutrition and health care (X)<br>5. Respect among household members (G7)<br>6. Attitudes toward IPV (G9)

Table 2: Data collection timeline

Methods	Participants	timeline
Semi-structured in-depth interview at the household level		
In-depth interviews with women	1) Pregnant women<br>2) Mothers of children <1 month<br>3) Mothers 1-5 months<br>4) Mothers of children 6 – 59 months	➢ At the start of SCC Trial<br>➢ Endline
In-depth interviews with family members	1) Mother or mother-in-law of women<br>2) Husband of women	➢ At the start of SCC Trial<br>➢ Endline

For peer review only

For peer review only - http://bmjopen.bmj.com/site/about/guidelines.xhtml

	3) Other key family members as directed by the woman interviewed	
Pro-WEAI Survey questions		
Survey questionnaires	Female intervention recipients	➢ Baseline<br>➢ Endline

Table 3: Social Desirability Bias

**Directions: Read each item and decide whether it is true (T) or false (F) for you.**			
		**True**	**False**
1	It is sometimes hard for me to go on with my work if I am not encouraged.		
2	I sometimes feel resentful when I don't get my way		
3	On a few occasions, I have given up doing something because I thought too little of my ability		
4	There have been times when I felt like rebelling against people in authority even though I knew they were right		
5	No matter who I'm talking to, I'm always a good listener		
6	There have been occasions when I took advantage of someone		
7	I'm always willing to admit it when I make a mistake		
8	I sometimes try to get even rather than forgive and forget		
9	I am always courteous, even to people who are disagreeable		
10	I have never been irked when people expressed ideas very different from my own		
11	There have been times when I was quite jealous of the good fortune of others		
12	I am sometimes irritated by people who ask favours of me		
13	I have never deliberately said something that hurt someone's feelings		

For peer review only

For peer review only - http://bmjopen.bmj.com/site/about/guidelines.xhtml

SPIRIT

STANDARD PROTOCOL ITEMS: RECOMMENDATIONS FOR INTERVENTIONAL TRIALS

SPIRIT 2013 Checklist: Recommended items to address in a clinical trial protocol and related documents*

Section/item	Item No	Description	Addressed on page number
**Administrative information**			
Title	1	Descriptive title identifying the study design, population, interventions, and, if applicable, trial acronym	___1________
Trial registration	2a	Trial identifier and registry name. If not yet registered, name of intended registry	___20________
	2b	All items from the World Health Organization Trial Registration Data Set	____________
Protocol version	3	Date and version identifier	____________
Funding	4	Sources and types of financial, material, and other support	___20_______
Roles and responsibilities	5a	Names, affiliations, and roles of protocol contributors	___1 and 21_
	5b	Name and contact information for the trial sponsor	_____________
	5c	Role of study sponsor and funders, if any, in study design; collection, management, analysis, and interpretation of data; writing of the report; and the decision to submit the report for publication, including whether they will have ultimate authority over any of these activities	____22______
	5d	Composition, roles, and responsibilities of the coordinating centre, steering committee, endpoint adjudication committee, data management team, and other individuals or groups overseeing the trial, if applicable (see Item 21a for data monitoring committee)	_____________

For peer review only

Introduction

Background and rationale	6a	Description of research question and justification for undertaking the trial, including summary of relevant studies (published and unpublished) examining benefits and harms for each intervention	____4________
	6b	Explanation for choice of comparators	______________
Objectives	7	Specific objectives or hypotheses	____7 & 8_____
Trial design	8	Description of trial design including type of trial (eg, parallel group, crossover, factorial, single group), allocation ratio, and framework (eg, superiority, equivalence, noninferiority, exploratory)	____13 & 14__

Methods: Participants, interventions, and outcomes

Study setting	9	Description of study settings (eg, community clinic, academic hospital) and list of countries where data will be collected. Reference to where list of study sites can be obtained	____10_________
Eligibility criteria	10	Inclusion and exclusion criteria for participants. If applicable, eligibility criteria for study centres and individuals who will perform the interventions (eg, surgeons, psychotherapists)	____11_________
Interventions	11a	Interventions for each group with sufficient detail to allow replication, including how and when they will be administered	_____14_______
	11b	Criteria for discontinuing or modifying allocated interventions for a given trial participant (eg, drug dose change in response to harms, participant request, or improving/worsening disease)	______________
	11c	Strategies to improve adherence to intervention protocols, and any procedures for monitoring adherence (eg, drug tablet return, laboratory tests)	______________
	11d	Relevant concomitant care and interventions that are permitted or prohibited during the trial	______________
Outcomes	12	Primary, secondary, and other outcomes, including the specific measurement variable (eg, systolic blood pressure), analysis metric (eg, change from baseline, final value, time to event), method of aggregation (eg, median, proportion), and time point for each outcome. Explanation of the clinical relevance of chosen efficacy and harm outcomes is strongly recommended	____12_______
Participant timeline	13	Time schedule of enrolment, interventions (including any run-ins and washouts), assessments, and visits for participants. A schematic diagram is highly recommended (see Figure)	____13________

For peer review only - http://bmjopen.bmj.com/site/about/guidelines.xhtml

Sample size	14	Estimated number of participants needed to achieve study objectives and how it was determined, including clinical and statistical assumptions supporting any sample size calculations	____14_________
Recruitment	15	Strategies for achieving adequate participant enrolment to reach target sample size	____14_________

Methods: Assignment of interventions (for controlled trials)

Allocation:

Sequence generation	16a	Method of generating the allocation sequence (eg, computer-generated random numbers), and list of any factors for stratification. To reduce predictability of a random sequence, details of any planned restriction (eg, blocking) should be provided in a separate document that is unavailable to those who enrol participants or assign interventions	___14__________
Allocation concealment mechanism	16b	Mechanism of implementing the allocation sequence (eg, central telephone; sequentially numbered, opaque, sealed envelopes), describing any steps to conceal the sequence until interventions are assigned	___14_________
Implementation	16c	Who will generate the allocation sequence, who will enrol participants, and who will assign participants to interventions	___14__________
Blinding (masking)	17a	Who will be blinded after assignment to interventions (eg, trial participants, care providers, outcome assessors, data analysts), and how	___14__________
	17b	If blinded, circumstances under which unblinding is permissible, and procedure for revealing a participant's allocated intervention during the trial	_____________

Methods: Data collection, management, and analysis

Data collection methods	18a	Plans for assessment and collection of outcome, baseline, and other trial data, including any related processes to promote data quality (eg, duplicate measurements, training of assessors) and a description of study instruments (eg, questionnaires, laboratory tests) along with their reliability and validity, if known. Reference to where data collection forms can be found, if not in the protocol	____14_________
	18b	Plans to promote participant retention and complete follow-up, including list of any outcome data to be collected for participants who discontinue or deviate from intervention protocols	_____________

Data management	19	Plans for data entry, coding, security, and storage, including any related processes to promote data quality (eg, double data entry; range checks for data values). Reference to where details of data management procedures can be found, if not in the protocol	___17_________
Statistical methods	20a	Statistical methods for analysing primary and secondary outcomes. Reference to where other details of the statistical analysis plan can be found, if not in the protocol	____17_________
	20b	Methods for any additional analyses (eg, subgroup and adjusted analyses)	_____________
	20c	Definition of analysis population relating to protocol non-adherence (eg, as randomised analysis), and any statistical methods to handle missing data (eg, multiple imputation)	_____________
**Methods: Monitoring**			
Data monitoring	21a	Composition of data monitoring committee (DMC); summary of its role and reporting structure; statement of whether it is independent from the sponsor and competing interests; and reference to where further details about its charter can be found, if not in the protocol. Alternatively, an explanation of why a DMC is not needed	__17___________
	21b	Description of any interim analyses and stopping guidelines, including who will have access to these interim results and make the final decision to terminate the trial	_____________
Harms	22	Plans for collecting, assessing, reporting, and managing solicited and spontaneously reported adverse events and other unintended effects of trial interventions or trial conduct	____17_________
Auditing	23	Frequency and procedures for auditing trial conduct, if any, and whether the process will be independent from investigators and the sponsor	_____________
**Ethics and dissemination**			
Research ethics approval	24	Plans for seeking research ethics committee/institutional review board (REC/IRB) approval	___18__________
Protocol amendments	25	Plans for communicating important protocol modifications (eg, changes to eligibility criteria, outcomes, analyses) to relevant parties (eg, investigators, REC/IRBs, trial participants, trial registries, journals, regulators)	_____________

For peer review only - http://bmjopen.bmj.com/site/about/guidelines.xhtml

Consent or assent	26a	Who will obtain informed consent or assent from potential trial participants or authorised surrogates, and how (see Item 32)	____18________
	26b	Additional consent provisions for collection and use of participant data and biological specimens in ancillary studies, if applicable	______________
Confidentiality	27	How personal information about potential and enrolled participants will be collected, shared, and maintained in order to protect confidentiality before, during, and after the trial	_____18_______
Declaration of interests	28	Financial and other competing interests for principal investigators for the overall trial and each study site	____21__________
Access to data	29	Statement of who will have access to the final trial dataset, and disclosure of contractual agreements that limit such access for investigators	____18__________
Ancillary and post-trial care	30	Provisions, if any, for ancillary and post-trial care, and for compensation to those who suffer harm from trial participation	______________
Dissemination policy	31a	Plans for investigators and sponsor to communicate trial results to participants, healthcare professionals, the public, and other relevant groups (eg, via publication, reporting in results databases, or other data sharing arrangements), including any publication restrictions	____17________
	31b	Authorship eligibility guidelines and any intended use of professional writers	______________
	31c	Plans, if any, for granting public access to the full protocol, participant-level dataset, and statistical code	______________
**Appendices**			
Informed consent materials	32	Model consent form and other related documentation given to participants and authorised surrogates	______________
Biological specimens	33	Plans for collection, laboratory evaluation, and storage of biological specimens for genetic or molecular analysis in the current trial and for future use in ancillary studies, if applicable	______________

*It is strongly recommended that this checklist be read in conjunction with the SPIRIT 2013 Explanation & Elaboration for important clarification on the items. Amendments to the protocol should be tracked and dated. The SPIRIT checklist is copyrighted by the SPIRIT Group under the Creative Commons "Attribution-NonCommercial-NoDerivs 3.0 Unported" license.

For peer review only - http://bmjopen.bmj.com/site/about/guidelines.xhtml

BMJ Open

Assessing the impact of a combined nutrition counselling and cash transfer intervention on women's empowerment in rural Bangladesh: A randomised control trial protocol.

Journal:	*BMJ Open*
Manuscript ID	bmjopen-2020-044263.R2
Article Type:	Protocol
Date Submitted by the Author:	21-May-2021
Complete List of Authors:	Kirkwood, Elizabeth; The University of Sydney Faculty of Medicine and Health, Sydney School of Public Health;<br>Dibley, Michael; The University of Sydney, School of Public Health<br>Hoddinott, John Frederick ; Cornell University,<br>Huda, Tanvir ; The University of Sydney, Sydney School of Public Health<br>Laba, Tracey; University of Technology Sydney, Centre for Health Economics Research and Evaluation<br>Tahsina, Tazeen; International Centre for Diarrhoeal Disease Research Bangladesh, Maternal and Child Health Division<br>Hasan, Mohammad; International Centre for Diarrhoeal Disease Research Bangladesh<br>Iqbal, Afrin; International Centre for Diarrhoeal Disease Research Bangladesh<br>Khan, Jasmin; International Centre for Diarrhoeal Disease Research Bangladesh<br>Ali, Nazia; International Centre for Diarrhoeal Disease Research Bangladesh<br>Ullah, Saad; International Centre for Diarrhoeal Disease Research Bangladesh<br>Goodwin, Nicholas; The University of Sydney<br>Muthayya, Sumithra; The Sax Institute<br>Islam, M; International Centre for Diarrhoeal Disease Research Bangladesh, Nutrition and Clinical Services Division<br>Ara, Gulshan; International Centre for Diarrhoeal Disease Research Bangladesh, NCSD<br>Agho, Kingsley ; Western Sydney University, School of Science and Health,<br>Arifeen, Shams E.; ICDDR B, Maternal and Child Health Division (MCHD)<br>Alam, Ashraful; University of Sydney, School of Public Health
**Primary Subject Heading**:	Public health
Secondary Subject Heading:	Global health
Keywords:	PUBLIC HEALTH, Protocols & guidelines < HEALTH SERVICES ADMINISTRATION & MANAGEMENT, Clinical trials < THERAPEUTICS

SCHOLARONE™
Manuscripts

I, the Submitting Author has the right to grant and does grant on behalf of all authors of the Work (as defined in the below author licence), an exclusive licence and/or a non-exclusive licence for contributions from authors who are: i) UK Crown employees; ii) where BMJ has agreed a CC-BY licence shall apply, and/or iii) in accordance with the terms applicable for US Federal Government officers or employees acting as part of their official duties; on a worldwide, perpetual, irrevocable, royalty-free basis to BMJ Publishing Group Ltd ("BMJ") its licensees and where the relevant Journal is co-owned by BMJ to the co-owners of the Journal, to publish the Work in this journal and any other BMJ products and to exploit all rights, as set out in our licence.

The Submitting Author accepts and understands that any supply made under these terms is made by BMJ to the Submitting Author unless you are acting as an employee on behalf of your employer or a postgraduate student of an affiliated institution which is paying any applicable article publishing charge ("APC") for Open Access articles. Where the Submitting Author wishes to make the Work available on an Open Access basis (and intends to pay the relevant APC), the terms of reuse of such Open Access shall be governed by a Creative Commons licence – details of these licences and which Creative Commons licence will apply to this Work are set out in our licence referred to above.

Other than as permitted in any relevant BMJ Author's Self Archiving Policies, I confirm this Work has not been accepted for publication elsewhere, is not being considered for publication elsewhere and does not duplicate material already published. I confirm all authors consent to publication of this Work and authorise the granting of this licence.

For peer review only

Author

Title

Assessing the impact of a combined nutrition counselling and cash transfer intervention on women's empowerment in rural Bangladesh: A randomised control trial protocol.

Authors

Elizabeth K. Kirkwood - ORCID 0000-0001-8603-4903

The University of Sydney, Sydney School of Public Health; Edward Ford Building, NSW, 2006, Australia.

elizabeth.kirkwood@sydney.edu.au

Michael J. Dibley - ORCID 0000-0002-1554-5180

The University of Sydney, Sydney School of Public Health; Edward Ford Building, NSW, 2006, Australia michael.dibley@sydney.edu.au

John Hoddinott – ORCID 0000-0002-0590-3917

Cornell University

Tanvir Huda – ORCID 0000-0002-8996-4361

The University of Sydney, Sydney School of Public Health; Edward Ford Building, NSW, 2006, Australia.

Tracey-Lea Laba – ORCID 0000-0002-5182-9092

University of Technology Sydney

Tazeen Tahsina – ORCID 0000-0001-5899-8422

International Centre for Diarrhoeal Disease Research, Bangladesh

Mohammad Mehedi Hasan – ORCID 0000-0003-0630-290X

International Centre for Diarrhoeal Disease Research, Bangladesh

Afrin Iqbal – ORCID 0000-0002-1859-8405

International Centre for Diarrhoeal Disease Research, Bangladesh

Jasmin Khan – ORCID 0000-0003-2022-6448

For peer review only

For peer review only - http://bmjopen.bmj.com/site/about/guidelines.xhtml

International Centre for Diarrhoeal Disease Research, Bangladesh

Nazia Binte Ali – ORCID 0000-0001-5010-9563

International Centre for Diarrhoeal Disease Research, Bangladesh

Saad Ullah - ORCID 0000-0001-7450-6483

International Centre for Diarrhoeal Disease Research, Bangladesh

Nicholas Goodwin – ORCID 0000-0003-2476-6310

The University of Sydney, Sydney School of Public Health; Edward Ford Building, NSW, 2006, Australia.

Sumithra Muthayya– ORCID 0000-0003-1645-9206

Sax Institute

Munirul Islam – ORCID 0000-0002-8780-8760

International Centre for Diarrhoeal Disease Research, Bangladesh

Gulshan Ara– ORCID 0000-0002-3745-5952

International Centre for Diarrhoeal Disease Research, Bangladesh

Kingsley Emwinyore Agho – ORCID 0000-0003-4111-3207

Western Sydney University

Shams El Arifeen – ORCID 0000-0002-5372-5932

International Centre for Diarrhoeal Disease Research, Bangladesh

Ashraful Alam - ORCID: 0000-0001-7034-1095

The University of Sydney, Sydney School of Public Health; Edward Ford Building, NSW, 2006, Australia

neeloy.alam@sydney.edu.au

Corresponding author

Elizabeth K. Kirkwood

For peer review only

For peer review only - http://bmjopen.bmj.com/site/about/guidelines.xhtml

The University of Sydney, Sydney School of Public Health; Room 328 Edward Ford Building, NSW, 2006, Australia. Phone: +61 410 517 874

Email: elizabeth.kirkwood@sydney.edu.au

For peer review only

For peer review only - http://bmjopen.bmj.com/site/about/guidelines.xhtml

Abstract

Introduction: There is growing interest in assessing the impact of health interventions, particularly when women are the focus of the intervention, on women's empowerment. Globally, research has shown that interventions targeting nutrition, health, and economic development can affect women's empowerment. Evidence suggests that women's empowerment is also an underlying determinant of nutrition outcomes. Depending on the focus of the intervention, different domains of women's empowerment will be influenced for example an increase in nutritional knowledge, or greater control over income and access to resources.

Objective: This study evaluates the impact of the Shonjibon Cash and Counselling trial that combines nutrition counselling and unconditional cash transfer intervention, delivered on a mobile platform, on women's empowerment in rural Bangladesh.

Methods and analysis: We will use a mixed-methods approach, combining statistical analysis of quantitative data from 2,080 women in a cluster randomised controlled trial examining the impact of nutrition behaviour change communications and cash transfers on child undernutrition. Pregnant participants will be given a smartphone with a customised app, delivering nutrition behaviour change communication messages, receive nutrition counselling via a call centre and an unconditional cash transfer. This study is a component of the Shonjibon Cash and Counselling trial and will measure women's empowerment using a composite indicator based on the Project-Level Women's Empowerment in Agriculture Index, with quantitative data collection at baseline and endline. Thematic analysis of qualitative data, collected through longitudinal interviews with women, husbands, and mothers-in-law, will elicit a local understanding of women's empowerment and the linkages between the intervention and women's empowerment outcomes. This paper describes the study protocol to evaluate women's empowerment in a nutrition-specific and sensitive-intervention using internationally validated, innovative

For peer review only

tools and will help fill the evidence gap on pathways of impact, highlighting areas to target for future programming.

Ethics and dissemination:

Ethical approval has been obtained from the International Centre for Diarrhoeal Disease Research (Ref. PR 17106) and The University of Sydney (Ref: 2019/840). Findings from this study will be shared in Bangladesh with dissemination sessions in-country, internationally at conferences, and published in peer-reviewed journals.

Word count: 5325

Keywords

Women's empowerment, nutrition, cash transfer, counselling, BCC, mHealth, protocol, Bangladesh.

Strengths and limitations of this study

- This paper describes the study protocol evaluating women's empowerment in a nutrition-specific and -sensitive RCT and will help fill the evidence gap on pathways of impact for women's empowerment and highlight areas to target for future policy and programming.
- Formative research guided this mixed-methods approach.
- We have designed specifically tailored tools, based on a theory of change, and utilising an internationally validated index, that has been piloted in Bangladesh.
- We are not using the Pro-WEAI in full as we interview women only for the quantitative component and will not calculate the Gender Parity Index, which index sheds light on the sense of intrahousehold inequality, however, we will explore this with qualitative research.

Background

To define women's empowerment is not straightforward, nor is it easy to measure across the different domains and phases of a woman's life. Empowerment is "the process of enhancing an individual's or group's capacity to make purposive choices and to transform those choices into desired actions and outcomes"(1). The United Nations defines women's empowerment as having five key components:

For peer review only - http://bmjopen.bmj.com/site/about/guidelines.xhtml

For peer review only

"women's sense of self-worth; their right to have and to determine choices; their right to have access to opportunities and resources; their right to have the power to control their own lives, both within and outside the home; and their ability to influence the direction of social change to create a more just social and economic order, nationally and internationally" (2).

Gender, socially constructed roles assigned by sex, is one of the keynote determinants of health outcomes (3), making gender equality and the rights of women an imperative goal in global public health. Gender inequality and restrictive gender norms are, according to the Lancet 2019 series on Gender Equality, Norms and Health "powerful but separate determinants of health and well-being" (3). Sustainable Development Goal (SDG) 5 aims to achieve gender equality and empower all women and girls, ending discrimination in all forms; from violence to forced marriage, full active participation and equal rights in all spheres of life – politically, economically, and socially (4). Out of the 17 SDGs, 11 entail indicators related to gender.

Much progress has been made toward gender equality and the empowerment of women globally. Yet, the potential for women to fully participate as "agents of change" is still limited due to persistent social, economic and political inequalities (5). Women continue to be constrained by norms, beliefs, and customs through which societies differentiate between women and men (6). Contextual forms of oppression include patriarchal societies and institutions, as well as lack of opportunity due to socioeconomic circumstances such as poverty. Violence, including intimate partner violence (IPV), disproportionally affects women. Worldwide, a third of women report experience physical or sexual IPV (7). Women subjected to IPV suffer poor mental and physical health, and their children show poorer health, nutrition and development outcomes (8). Evidence suggests that the combination of cash transfers and behaviour change communication (BCC), can increase women's bargaining power and poverty-related emotional well-being and lead to a reduction in IPV (8).

For peer review only - http://bmjopen.bmj.com/site/about/guidelines.xhtml

113 Interventions to improve the health and nutrition literacy of women and, in particular, counselling
114 through mobile phone applications can make a positive impact on women's empowerment. Mobile health
115 (mHealth) interventions have the potential to improve a woman's confidence and decision-making skills
116 in communicating with health care professionals. However, a systematic review revealed the need for a
117 further rigorous investigation into mHealth, in terms of implementation and evaluation, to establish
118 whether mHealth programs transform rather than reinforce gender inequalities (9). When a woman
119 receives additional resources, such as cash transfers and is the target of a mHealth program this can
120 challenge gender norms within relationships and exacerbate gender disparities (9). There is limited
121 evidence on the adverse effects of mHealth, such as expanding the digital divide and gender-based power
122 imbalances, and this lack of evidence emphasises the need to monitor the impact of mHealth
123 interventions on gender relations and women's empowerment (9).

124 The Lancet Series on Maternal and Child nutrition also advocates the use of cash transfer and social safety
125 net programmes, which are increasingly the preferred approach to support households living in chronic
126 and extreme poverty (10). When targeted at women, social protection can promote women's economic
127 empowerment and enhance decision-making ability, with the overarching assumption that control over
128 cash will lead to greater investment in children's health and education (10, 11). When women are engaged
129 in nutrition-sensitive programmes that utilise social safety nets, certain aspects of women's
130 empowerment are augmented, such as changes in gender roles and intrahousehold bargaining power
131 (10).

132 We plan to conduct a study, embedded in a cluster randomised control trial that assesses a multifaceted
133 nutrition intervention on childhood stunting - Shonjibon Cash and Counselling Trial (SCC) (12). This
134 protocol paper presents the way we will measure the impact of the SCC trial women's empowerment.

135 **Developmental interventions and women's empowerment in Bangladesh**

For peer review only - http://bmjopen.bmj.com/site/about/guidelines.xhtml

Although Bangladesh has made remarkable reductions in rates of poverty, almost 1 in 4 of the population still live in poverty (24.3% in 2016) (13). In 2018, the agriculture sector accounted for 40% of total employment (14). In most developing countries, women account for almost half of the agricultural labour force; however, in Bangladesh, women exceed fifty per cent of the agricultural labour force (15). In rural Bangladesh, social constructs such as gender roles of women and men powerfully drive household food consumption (16). A nationally representative sample from Bangladesh found that women's empowerment increases dietary diversity and availability of calories at a household level (17, 18). The Government of Bangladesh and development organisations have shown a keen interest in combining nutrition counselling and cash transfers to improve maternal and child nutrition (19).

In rural Bangladesh, 84% of households have access to a mobile phone (20). A study in Bangladesh revealed women using Aponjon, a mobile-messaging based service providing information about mother and babies, had higher rates of autonomy in accessing mobile phones, although two-thirds of the women resided in urban areas. mHealth consultations may help address socio-cultural gender norms, and empower women as they were able to discuss personal health issues on the phone with female doctors who are infrequently found in rural communities (20). The need to collect sex aggregated, valid, comprehensive, and standardised empowerment data is therefore imperative to achieve not only SDG 5 but to enable the consistent measurement of women's empowerment indices (21, 22).

Objectives and Hypotheses

This study aims to evaluate the impact of a combined nutrition counselling and unconditional cash transfer intervention (Shonjibon Cash and Counselling / SCC Trial), delivered on a mobile platform, on the level of empowerment of women in rural Bangladesh. This study will assess women's experience of empowerment and disempowerment throughout the SCC trial.

For peer review only - http://bmjopen.bmj.com/site/about/guidelines.xhtml

The **primary hypothesis** is that in a community-based cluster randomised control trial of a mobile phone-based nutrition behaviour change communication (BCC) and unconditional cash transfer, given to women in low-income families in rural Bangladesh, women's empowerment will increase as measured by an increase in mean women's empowerment scores (by an average percentage of 20) from the baseline to the end of the 24-month intervention, compared to women in the control arm.

The **secondary hypotheses** are that the mobile phone nutrition BCC and unconditional cash transfer compared to usual programs will: a) increase control over income and economic resources; b) improve input and decision-making power in nutrition and health care choices; c) decrease the acceptance and experience of intimate partner violence.

SCC Trial women's empowerment Theory of Change

Empowering women is not an explicit goal of the SCC Trial; however, women gain access to nutrition counselling, cash transfers and a mobile phone; the utilisation of these resources can empower women. We constructed the theory of change to illustrate how the SCC Trial will impact on women's empowerment outcomes. It outlines the barriers, intervention details, intermediate outcomes, outcomes and impact pathways (Figure 1).

The SCC Trial intervention is delivered on a mobile platform. Women receive an mHealth interactive app – messages, audio and video, quizzes – as well as counselling from the call centre. They also receive cash via BKash mobile banking app. The principal rationale of the theory of change causal pathway to impact is that SCC Trial will lead to an increase in women's empowerment for those women participating in the intervention arm.

The Project-level Women's Empowerment in Agriculture Index (Pro-WEAI)(21) indicators, discussed in detail later, appear in the theory of change across the outcomes. We will use the six indicators from the Pro-WEAI to calculate the women's empowerment index and include: 1) Control over use of income; 2)

For peer review only - http://bmjopen.bmj.com/site/about/guidelines.xhtml

Input into productive decisions; 3) Autonomy in income; 4) Decision-making power on nutrition and health care; 5) Respect among household members; 6) Attitudes toward IPV.

One of the barriers to nutrition faced in rural Bangladesh is limited access to nutrition and infant and young child feeding (IYCF) information. Women in the SCC Trial intervention arm receive nutrition, IYCF and livelihood BCC delivered on a mobile app and nutrition counselling from the call centre. The intermediate outcome is woman's acquisition of new BCC/nutrition knowledge, contributing to increased input into and decision-making power about health and nutrition choices for herself and her family (Indicator 4). Improvements in communication also occur (spousal, intrahousehold, community) as new knowledge is discussed and shared, including ways to put the messages into action (Indicator 5). One's ability to make decisions is a measure of empowerment (23). New knowledge can be used as leverage and bargaining power and enhance decision-making abilities across a range of areas such as livelihoods, household expenditure and decisions on productive assets, i.e., what crops to farm (Indicator 2,3,4).

The SCC Trial setting is in one of the poorest regions in Bangladesh. Families face barriers such as food insecurity and lack of financial resources. When a woman is the recipient of a cash transfer, the intermediate outcome is increased control over the use of income (Indicator 1). Cash transfers, targeted at women, have the potential to improve livelihoods, alleviate poverty, enhance food security and increase women's empowerment (10). Spousal communication is also strengthened as discussion ensues as to how to spend the additional income (Indicator 4). The outcomes for women receiving an unconditional cash transfer include a strengthened ability to contribute to and participate in financial decision making. This decision-making covers health and nutrition choices, household expenditure, productive assets, livelihood choices (i.e. homestead gardening, buying chickens to raise for eggs, meat and income) (Indicator 1,2,3,4).

For peer review only - http://bmjopen.bmj.com/site/about/guidelines.xhtml

IPV includes behaviour that is physical, psychological, sexual or abusive or controlling in nature (24). There is evidence that the combination of additional income from cash transfers combined with BCC, can increase women's bargaining power and poverty-related emotional well-being (8). Changing attitudes, from within the family unit, as women participate and learn from intervention activities has the potential to lead to a change in the acceptability of IPV, linked to a woman's right to bodily integrity (25). Women may also experience an increase in respect and self-efficacy and subsequent reduction in acceptance of IPV (Indicator 6).

Figure 1 SCC Trial women's empowerment Theory of Change (adapted from De Silva et al. (26)

Study Design

We will embed this study on women's empowerment within the SCC Trial. The SCC Trial will recruit pregnant women and follow up over 24 months an expected 2,080 mother-child dyads, from recruitment in early pregnancy until the child is 18 months of age. The intervention will run for 36 months, depending on the time of enrolment and gestation, and age of the child.

Methods

This study will use a mixed-methods approach with data gathered on women's empowerment from participants in the SCC Trial. This protocol paper will adhere to the SPIRIT and Tidier guidelines for clinical trial protocols (27-29) to ensure rigour in protocol content.

Study setting

We will conduct the study in two subdistricts of Sirajganj; Ullapara and Kamarkhanda, in northern Bangladesh (Figure 2). Employment in Sirajganj is predominantly in agriculture (51 %) with 25 % working in services and 23% in industry (30).

Figure 2: Sirajganj, Bangladesh SCC Study site

For peer review only

For peer review only - http://bmjopen.bmj.com/site/about/guidelines.xhtml

Eligibility criteria

All married women between 15-49 years, that are permanent residents of the study area and provide consent to participate and test positive with pregnancy urine test kit (Excel®) (whose gestational age is ≤90 days) are eligible to participate in SCC Trial.

Intervention: Shonjibon[1] Cash and Counselling Trial

A separate protocol paper provides details of the design of the SCC Trial (12). Briefly, the SCC Trial aims to assess the effectiveness of nutrition behaviour change communication (delivered via call centre counselling and specifically tailored mobile app) combined with unconditional cash transfers in reducing the prevalence of stunting (length-for-age < -2 Z) in children at 24 months. We will use a longitudinal cluster randomised controlled trial to determine the effectiveness of unconditional cash transfers and mobile behaviour change communications (BCC) to reduce child undernutrition in Sirajganj district in northern Bangladesh, one of the poorest regions in the country according to the World Food Program Poverty Maps (31). Both quantitative and qualitative approaches will be employed to address the study objectives.

The SCC Trial intervention arm receives: 1) nutrition BCC delivered on a specially tailored app on a smartphone (audio, video, and animation), 2) direct nutrition counselling from a call centre and 3) unconditional cash transfer of 1000 Taka (USD 12.50) received monthly via BKash mobile banking app. The control arm will receive a mobile phone and the current Government of Bangladesh health and nutrition services. The participants in the control arm receive a mobile phone as the trial is interested in the effects of the messages, nutrition counselling and cash transfer delivered on a mobile platform, as opposed to the non-specific effects of a mobile phone.

[1] [1]Shonjibon stands for Shustho Notun Jibon in Bangla, translated, it means "Healthy New Life"

For peer review only - http://bmjopen.bmj.com/site/about/guidelines.xhtml

Study outcomes

The primary study outcome will be the difference in women's empowerment scores in the intervention arm as compared to the control arm of the SCC Trial. Secondary study outcomes will include 1) control over income and economic resources; 2) input and decision-making power in nutrition and health care choices; 3) experience and attitude toward intimate partner violence.

Outcome measurements

The SCC trial will collect data across the main module of the Pro-WEAI (12 indicators) as well as the additional health and Pro-WEAI Nutrition and Health module. However, 6 of the Pro-WEAI indicators are likely to be directly impacted by the SCC trial, therefore, we will develop a composite women's empowerment indicator based on the following: 1) control over the use of income; 2) input in production decisions; 3) autonomy in income; 4) decision making power about women and children's health and nutrition; 5) respect among household members and 6) attitudes toward IPV (Appendix Table 1). We will also measure women's and family member's perceived and lived experiences related to women empowerment qualitatively (Appendix Table 1).

1. ***Control over the use of income***

We will measure control over the use of income by asking questions from Pro-WEAI; these questions are significant as the SCC Trial gives an unconditional cash transfer to the woman. Quantitative questions assess a woman's input into decision making on all sources of household income. Qualitative interviews will explore local perceptions of intrahousehold harmony and communication skills, issues related to withdrawing the cash, and perceptions and experience of control and expenditure of the money.

2. ***Input in productive decisions***

For peer review only - http://bmjopen.bmj.com/site/about/guidelines.xhtml

These Pro-WEAI questions centre around input into household decision making on areas such as livelihood choices (activities the household participate in such as agricultural activities, home gardens etc), employment and household purchases.

3. Autonomy in income

A series of vignettes, validated by cognitive testing, using a series of hypothetical scenarios to assess motivation as to how income is used.

4. Decision-making ability about health and nutrition choices

We will examine responses from the Pro-WEAI Nutrition and Health Module regarding confidence and participation in decisions about nutrition and infant feeding, as well as a range of issues affecting women and children such as whether to consult a doctor when ill to dietary choices when pregnant.

5. Respect among household members

Questions in this indicator pertain to a woman's spouse or other household members on respect and trust within relationships, and level of comfort in disagreeing with household members.

6. Intimate Partner Violence

Previous research in Bangladesh has found that cash transfers and behaviour change communication can reduce IPV (8). We will measure a woman's attitude toward IPV using the Pro-WEAI as well as the direct experience of IPV using the questions from the Violence Against Woman and Girls 2015 Survey (32, 33). We will also conduct in-depth interviews to explore the woman's attitudes and perceptions of IPV qualitatively.

Participant recruitment and timeline

For peer review only

287 A household census in the study area will record the names and contact details of all women that agree to participate, and we will enter their details into an electronic system. The system will generate for each participant with a unique ID number and quick response (QR) code. SCC surveillance workers will then conduct door-to-door visits each month to identify women that have missed two menstrual periods in a row. The woman will then undertake a pregnancy test (using a urine test kit (Excel®). We will invite women who test positive for pregnancy to participate in the study with appropriate informed written consent. Based on experience with our pilot study, we anticipate 95% of the mothers will consent. Qualitative and quantitative data will be gathered at the start of the intervention and at endline (Appendix Table 2).

Allocation of clusters

Our study site has over 1100 villages, which would be adequate to cover the anticipated sample size. Each cluster encompasses 2-4 villages and consists of ~1000 households and ~4500 population. We will use a fixed randomization scheme to assign treatments to eligible clusters, with a uniform allocation ratio of treatments, stratified by Union. The sample size will consist of 104 clusters, total women (N = 2840) allocated to two parallel groups (n = 1420) with 20 mother-infant dyads per cluster. Each cluster consists of 1-4 adjacent villages with a minimum population of 600 households per cluster with 454 villages in our study area for clustering. A subsample of the households from a purposively selected subset of clusters will be chosen for qualitative data.

The intervention and control groups will receive similar maternal and child health and nutrition services from the government, and the critical difference will be the nutrition behaviour change communications with cash transfers on a mobile platform received by women in the intervention arm. The study design will control for potential observed and unobserved confounding factors as there will be an adequate number of clusters randomly allocated to the treatment groups. The geographic separation of clusters will

For peer review only - http://bmjopen.bmj.com/site/about/guidelines.xhtml

limit the contamination of the intervention arm. We will use Stata® software to generate the random allocation sequence. We are unable to mask the treatment arm due to the nature of the intervention.

Sample Size and power

The sample size for our trial is fixed by the primary hypothesis in the main Shonjibon Cash and Counselling trial, which estimated a total sample of 2184 mother-infant pairs from 104 clusters (12). We can find no reports of intra cluster correlation coefficients (icc) for the women's empowerment indicators we plan to use. Therefore, we estimate that the fixed trial sample size will provide at least 80% power to detect a 20% increase in women's empowerment assuming 30% of women are empowered (ref) and a high icc of 0.2. Assuming a lower icc of 0.05 we will have 90% power to detect a 13% increase in women's empowerment.

Data Collection methods

The SCC Trial will collect key household data and nutrition indices, while this study collects data on women's empowerment using the Pro-WEAI, whose focus is measuring agency for which there are limited if any standardised measures (21). We will include the Pro-WEAI nutrition and health module and supplementary questions regarding intimate partner violence. The qualitative component will be tailored to context-specific issues and guided by formative research.

Quantitative data

We will collect the women's empowerment indicators in household surveys at the baseline and the endline of the 24-month study in the intervention and non-intervention arms. We will pre-test all questions before the commencement of the study. We will use the validated short version of Marlowe-Crowne's social desirability scale (34), which is based on a subset of 13 items from the original scale. We will calculate a social desirability score by adding up the number of socially desirable answers, out of the

For peer review only - http://bmjopen.bmj.com/site/about/guidelines.xhtml

331 13 questions. The potential range of the score will be from 0-13 and we will create three categories with
332 a score of 0-4 graded as a low score, 5-9 as a medium score, and 10-14 as a high score (Appendix Table 3).
333 The questionnaire assesses whether social desirability bias or the tendency of respondents to answer
334 questions in a way viewed favourably by the research team has influenced the data collected. We will
335 train the field researchers in detecting social desirability bias in qualitative data and limit bias by properly
336 introducing the study to the respondents, building rapport and asking context-specific probing questions
337 following the recent framework described by Bergen and Labonté (35). We will follow the process
338 throughout the research.

339 **Qualitative data**

340 The study uses qualitative analysis to elicit a local understanding of women's empowerment and the
341 linkages between the SCC Trial and women's empowerment outcomes. We will generate qualitative data
342 on women's and men's perceived and experienced change in empowerment through longitudinal
343 qualitative semi-structured in-depth interviews. We will also interview key family members as suggested
344 by the women, for example, husband or mother-in-law.

345 We will explore the myriad of ways that women and men perceive and describe empowerment in their
346 communities. A purposive sample will be interviewed approximately two months after the start of the
347 intervention and at the endline of the SCC Trial. Due to the highly sensitive nature of the topics, a separate
348 team of interviewers, from a different Upazila, will be trained to collect data on women's empowerment
349 and intimate partner violence. We will train the team in line with the ethical and safety procedures as per
350 the World Health Organization's guidelines (36). The training will cover measures such as changing topics
351 and asking a decoy nutrition question if anyone comes into the interview space and having details of
352 referral and support services for women affected by or experiencing IPV.

353 ***Pro-WEAI***

For peer review only - http://bmjopen.bmj.com/site/about/guidelines.xhtml

354 The Pro-WEAI measures the empowerment of women, agency and inclusion in the agriculture sector (21).
355 It is a standardised, survey-based internationally validated index, developed to adapt to project-specific
356 contexts and has been piloted in Bangladesh (17, 21). This study uses Kabeer's conceptual framework of
357 empowerment focuses on the ability of individuals to make choices, ''a process of change during which
358 those who have been denied the ability to make choices acquire such an ability'' (37, 38). Agency or the
359 ability of the individual to make considered choices is at the centre of the framework and revolves around
360 interrelated dimensions of resources, agency and achievements. This framework underpins the Pro-WEAI
361 (21). This study will collect data from women, across twelve indicators that are equally weighted (Figure
362 3), with the addition of the Project-specific Nutrition and Health module.

363 *Figure 3: Pro-WEAI 3 Domains of Empowerment and 12 Indicators (21)*

364 ***Intimate Partner Violence***

365 The Pro-WEAI includes questions on a woman's attitude to intimate partner violence (IPV). We will collect
366 additional data about personal experience and behaviour specific questions regarding IPV, as opposed to
367 her perceptions thereof. We took these questions from the WHO Violence Against Women and Girls
368 Survey (Bangladesh 2015) (33). We will also ask questions relating to cyberviolence (39).

369 ***Health and Nutrition***

370 The Pro-WEAI offers the option of adding project-specific modules, and for this study, we will add the
371 Nutrition and Health Module. This module asks questions about decision making about nutrition and
372 health indices for women and children.

373 **Data collection and management**

374 Intervention and evaluation staff will be separate to and not know the trial hypothesis to minimise
375 assessment bias. Android Tablets will be used in the field to enable interactive data collection. During each

For peer review only - http://bmjopen.bmj.com/site/about/guidelines.xhtml

376 evaluation interview, the data collectors will be guided and navigated by the software. As soon as the field staff complete each interview, they will upload the data to the server at icddr,b. The data will be retained on the tablet and copied/merged with files at icddr,b., with android tabs being synchronised with the server daily. We will systematically scrutinise the captured data and address any discrepancies as they occur. Qualitative narrative data will be captured in digital audio recorders and stored in the server from where we will check for quality and improvement for future interviews.

Quantitative Data Analysis

Data analysis will be by intention to treat. The women's empowerment composite index scores will be categorized as the percentage of women as empowered or disempowered. For each empowerment indicator individuals are classified as adequate or inadequate based on Pro-WEAI predetermined thresholds. Women are considered empowered if 4 out of 6 indicators are adequate. We will also analyze impact of SCC intervention on individual indicators to assess increases or decreases in empowerment as not all indicators will respond or be impacted in the same way.

Analyses will be conducted at the mother-infant dyad level but will be adjusted for the cluster randomization (40). Primary analyses will compare the prevalence of women's empowerment at the end of the trial using Pearson's chi-square tests and 95% confidence intervals for the group difference, adjusted for clustering and generalized linear mixed models for non-continuous outcomes (e.g. logistic mixed models for binary outcomes e.g. percentage of women's empowerment). Models will include treatment group as a fixed effect, infants as a random effect to account for repeated measurements, and community-cluster as a random effect to account for cluster effects. We will also assess if the women's empowerment level at baseline, age, education and presence or absence of mother-in-law in the household modify the empowerment response to the intervention by testing for interactions between the intervention and these factors. STATA® will be used for all analyses.

For peer review only - http://bmjopen.bmj.com/site/about/guidelines.xhtml

399 **Qualitative Data Analysis**

400 We will record all interviews using digital audio recording devices. Interviews will be transcribed verbatim
401 in Bangla by the research team at icddr,b. We will maintain quality control, by checking the transcripts at
402 random intervals, to ensure the quality and accuracy of transcriptions. Data will be coded using thematic
403 analysis approach (41). We will use NVivo software to organise, code, categorise and compile the data.
404 Qualitative methods offer an effective way of describing in-depth, complex and varied perceptions of
405 empowerment within the local context. They will also aid with the interpretation of individual indicators
406 within the quantitative data. Data triangulation by using different sources of data will add strength to the
407 analysis (42).

408 **Data safety and monitoring board**

409 An independent data and safety monitoring board (DSMB) will be formed to assess the completeness
410 and quality of data and to ensure data is compliant with recruitment and retention goals. The DSMB will
411 also assess any factors that might affect the study outcome or compromise the confidentiality of the
412 trial data. Any unintended effects of the trial will be reported to the board.

413 **Patient and Public Involvement**

414 The development of the research question and outcome measures was informed by participants'
415 priorities, experience, and preferences based on our formative research and pilot study, whereby we
416 engaged the local community and sought their views about the proposed intervention. Participants were
417 involved in the design of the study by taking part in the formative research. Participants were not involved
418 in the recruitment and conduct of the study. The key trial findings will be disseminated to study
419 participants through meetings with community gatekeepers and local administration. In this RCT, the
420 burden of the intervention assessed by participants themselves as we undertook a pilot study.

For peer review only - http://bmjopen.bmj.com/site/about/guidelines.xhtml

Dissemination plan

Lessons learned from this study will be shared in Bangladesh with dissemination sessions in-country. Internationally, key findings will be shared with stakeholders, results will be presented at conferences, and published in international peer-reviewed journals.

Access to data

The SCC investigators will have access to all data and the right to analyse and publish data. Datasets will be shared after all personally identifiable information has been removed and to keep the identification of study subjects in confidence. The datasets generated and analysed during the study will be available from the corresponding author on reasonable request.

Ethics approval and consent

We have obtained approval for the SCC Trial from the Ethical Review Committee (ERC) of the International Centre for Diarrhoeal Disease Research (icddr,b) – (Ref. PR 17106), and the Human Research Ethics Committees (HRECs) at The University of Sydney (Ref: 2019/840).

We will train the women on the use of smartphones as well as the nutrition counselling application. We will store the data obtained on the use of the application with access to limited authorised project staff. Trained field staff will educate the women about the safety procedures for receipt and use of money. We will record responses anonymously and by identification number. We will maintain the confidentiality, privacy, and anonymity of the participants.

Project field workers will explain the nature, purpose, benefits, risks, and process of the trial to potential participants as they register themselves in the surveillance system if they meet the inclusion criteria. We will train them to obtain written informed consent (in Bangla) from all intervention participants. Participation in the research will be voluntary, and the respondent will have every right to withdraw

For peer review only

For peer review only - http://bmjopen.bmj.com/site/about/guidelines.xhtml

anytime throughout the programme without any obligation, loss, or penalty. We will explain the participant's rights before collecting any data.

Discussion

The main goal of this study is to evaluate the impact of a combined nutrition counselling and unconditional cash transfer intervention, delivered on a mobile platform, on women's empowerment in rural Bangladesh. We recognise that women's empowerment is an essential goal in global public health and the key to sustainable development. It is therefore crucial, that in complex and large-scale nutrition interventions, such as the SCC Trial, that we measure pathways of impact across the domains of women's empowerment. The 2013 Lancet Series on Maternal and Child Nutrition proposed utilising interventions that raise women's overall level of empowerment (10). Research has shown evidence of the impact of various development interventions on women's empowerment (43-45). A 2018 analysis of surveys from 54 countries observed that in critical aspects of family relationships, four out of five women did not hold agency (46). Cunningham et al. (2015) (47) note that women lack not only the resources to make significant decisions leading to better health and nutritional outcomes, but when disempowered, they also lack the autonomy and decision-making power within the household to make these critical decisions, having a multiplier effect on family and community.

This study has several strengths; firstly, it is embedded within a cluster RCT that aims to assess the effectiveness of a nutrition behaviour change communication combined with unconditional cash transfers in reducing the prevalence of stunting. Another strength lies in the use of a mixed-methods approach – obtaining qualitative and quantitative data. We gain further advantage from the use of tools tailored specifically for this intervention; tools that are based on formative research, wide-ranging literature review and piloted in Bangladesh, providing accurate, comprehensive and appropriate methodology.

For peer review only - http://bmjopen.bmj.com/site/about/guidelines.xhtml

The Pro-WEAI is designed to assess the impact evaluation of agricultural development projects (48). The SCC Trial is not an agricultural intervention, however, women in Bangladesh characteristically do postharvest activities and processing yet do not classify themselves as agricultural workers. It is in this context that we are using the index, to survey women in Bangladesh, whose livelihoods are bound to the agricultural sector (49). Alkire (2018) describes the Pro-WEAI as an "information platform" that is applicable in broader contexts other than exclusively agricultural interventions (50). The core elements of the SCC women's empowerment index, a customised tool, will provide translatable results – using measures and indices that allow global comparisons.

When the Pro-WEAI is used in full – women and men are interviewed - this enables the calculation of the Gender Parity Index, which measures empowerment and disempowerment within the household. The limitation of not interviewing men is that we may not capture the sense of intrahousehold inequality - are the women at least as empowered as the men in their households? We will account for this by ensuring that qualitative exploration delves into the extent to which the disempowerment observed is attributable to gender norms versus other causes. The SCC Trial does not directly focus on women's empowerment nor contest existing gender norms or patriarchal power structures. The study participants - the recipients of the cash transfer, nutrition counselling and smartphone - are, however, women. This study will identify where gaps in empowerment occur, highlight key areas of disempowerment and which domains help facilitate or hinder nutrition outcomes.

Funding

The Shonjibon Cash and Counselling Trial is funded by the National Health and Medical Research Council of Australia GNT 1120507. EKK received seed funding for a field visit from the University of Sydney Faculty of Medicine and Health. The funders do not have any role in the study design, data collection, and interpretation of data.

For peer review only - http://bmjopen.bmj.com/site/about/guidelines.xhtml

488 **Trial registration:**

489 • The Shonjibon Cash and Counselling Trial is registered with the Australian New Zealand Clinical
490 Trials Registry (ANZCTR) - The registration ID is ACTRN 12618001975280.

491 **Abbreviations**

492 • BCC behaviour change communication
493 • DSMB Data Safety Monitoring Board
494 • FGD focus group discussions
495 • icddr,b International Centre for Diarrhoeal Disease Research
496 • IDI in-depth-interview (IDI)
497 • IFPRI International Food Policy Research Institute
498 • IPV intimate partner violence
499 • IYCF infant young child feeding
500 • NHMRC National Health and Medical Research Council of Australia
501 • Pro-WEAI Project-Level Women's Empowerment in Agriculture Index
502 • SCC Trial Shonjibon Cash and Counselling Trial

503 **Author's contributions**

504 Elizabeth K Kirkwood drafted the paper, designed the study tools and main conceptual ideas. Ashraful
505 Alam and Michael Dibley made continuous contributions and supervised in the design of the study and
506 throughout the writing. All other authors (JH, TH, TLL, TT, MMH, AI, JK, NBA, SU, NG, SM, MI, GA, KEA, and
507 SA were involved in developing the main study design and methods and read, critically revised, and
508 approved the final manuscript, and met the ICMJE criteria for authorship.

509 **Acknowledgements**

For peer review only

For peer review only - http://bmjopen.bmj.com/site/about/guidelines.xhtml

We gratefully acknowledge the team at the International Food Policy Research Institute for their assistance and sharing the Project-Level Women's Empowerment in Agriculture Index. I would also like to thank the seminar attendees at the Sydney School of Public Health's Global Health and Nutrition Research Collaboration for providing valuable feedback at my presentation on the tools and methodology used in this paper.

Competing Interests

The authors declare that they do not have any competing interests.

For peer review only

For peer review only - http://bmjopen.bmj.com/site/about/guidelines.xhtml

References

1. Alsop R, Bertelsen MF, Holland J. Empowerment in practice - from analysis to implementation Washington, DC 20433 USA; 2006.

2. Department of Economic and Social Affairs UPD. Guidelines on Women's Empowerment. New York, USA: United Nations; 2010.

3. Heise L, Greene ME, Opper N, Stavropoulou M, Harper C, Nascimento M, et al. Gender inequality and restrictive gender norms: framing the challenges to health. The Lancet. 2019;393(10189):2440-54.

4. Transforming Our World: The 2030 Agenda for Sustainable Development. . United Nations, New York, USA.: United Nations; 2015. Contract No.: 09/10/2018.

5. The Future We Want. Rio + 20, Rio de Janeiro, Brazil: United Nations; 2012.

6. Kabeer N. The Power to Choose: Bangladeshi Women and Labour Market Decisions in London and Dhaka: Verso Books; 2000.

7. Global and regional estimates of violence against women: prevalence and health effects of intimate partner violence and non-partner sexual violence. Geneva: World Health Organization; 2013.

8. Roy S, Hidrobo M, Hoddinott J, Ahmed A. Transfers, Behavior Change Communication, and Intimate Partner Violence: Postprogram Evidence from Rural Bangladesh. The Review of Economics and Statistics. 2019;101(5):865-77.

9. Jennings L, Gagliardi L. Influence of mhealth interventions on gender relations in developing countries: a systematic literature review. International Journal for Equity in Health. 2013;12(1):85.

10. Ruel MT, Alderman H. Nutrition-sensitive interventions and programmes: how can they help to accelerate progress in improving maternal and child nutrition? The Lancet. 2013;382(9891):536-51.

11. Campos APdlO. Empowering rural women through social protection. Rome: FAO - Food and Agriculture Organization of the United Nations

2015.

12. Huda TM, Alam A, Tahsina T, Hasan MM, Iqbal A, Khan J, et al. Shonjibon cash and counselling: a community-based cluster randomised controlled trial to measure the effectiveness of unconditional cash transfers and mobile behaviour change communications to reduce child undernutrition in rural Bangladesh. BMC Public Health. 2020;20(1):1776.

13. Headey D, Hoddinott J, Ali D, Tesfaye R, Dereje M. The Other Asian Enigma: Explaining the Rapid Reduction of Undernutrition in Bangladesh. World Development. 2015;66:749-61.

14. Employment in agriculture (% of total employment) (modeled ILO estimate) [Internet]. 2018 [cited 13/05/2019]. Available from: https://data.worldbank.org/indicator/SL.AGR.EMPL.ZS?locations=BD.

15. Women Farmers of Bangladesh. Food and Agriculture organization of the United Nations; 2016.

16. Patalagsa MA, Schreinemachers P, Begum S, Begum S. Sowing seeds of empowerment: effect of women's home garden training in Bangladesh. Agriculture & Food Security. 2015;4(1):24.

17. Alkire S, Meinzen-Dick R, Peterman A, Quisumbing A, Seymour G, Vaz A. The Women's Empowerment in Agriculture Index. World Development. 2013;52:71-91.

18. Sraboni E, Malapit HJ, Quisumbing AR, Ahmed AU. Women's Empowerment in Agriculture: What Role for Food Security in Bangladesh? World Development. 2014;61:11-52.

19. Huda TM, Alam A, Tahsina T, Hasan MM, Khan J, Rahman MM, et al. Mobile-Based Nutrition Counseling and Unconditional Cash Transfers for Improving Maternal and Child Nutrition in Bangladesh: Pilot Study. JMIR mHealth and uHealth. 2018;6(7):e156-e.

For peer review only

For peer review only - http://bmjopen.bmj.com/site/about/guidelines.xhtml

20. Alam M, Banwell C, Olsen A, Lokuge K. Patients' and Doctors' Perceptions of a Mobile Phone–Based Consultation Service for Maternal, Neonatal, and Infant Health Care in Bangladesh: A Mixed-Methods Study. JMIR Mhealth Uhealth. 2019;7(4):e11842.
21. Malapit HJ, Quisumbing AR, Meinzen-Dick RS, Seymour G, Martinez EM, Heckert J, et al. Development of the Project-Level Women's Empowerment in Agriculture Index (Pro-WEAI). IFPRI; 2019 January 18, 2019.
22. Weber AM, Cislaghi B, Meausoone V, Abdalla S, Mejía-Guevara I, Loftus P, et al. Gender norms and health: insights from global survey data. The Lancet. 2019;393(10189):2455-68.
23. Seymour G, Peterman A. Context and measurement: An analysis of the relationship between intrahousehold decision making and autonomy. World Development. 2018;111:97-112.
24. Violence against women. World Health Organization; 2017.
25. Malapit H, Quisumbing A, Meinzen-Dick R, Seymour G, Martinez EM, Heckert J, et al. Development of the project-level Women's Empowerment in Agriculture Index (pro-WEAI). World Development. 2019;122:675-92.
26. De Silva MJ, Breuer E, Lee L, Asher L, Chowdhary N, Lund C, et al. Theory of Change: a theory-driven approach to enhance the Medical Research Council's framework for complex interventions. Trials. 2014;15(1):267.
27. Hoffmann Tammy C GPP, Boutron Isabelle, Milne Ruairidh, Perera Rafael, Moher David, Virginia Barbour, Marie Johnston, Sarah E. Lamb, Mary Dixon-Woods, Jeremy C. Wyatt. Better reporting of interventions: template for intervention description and replication (TIDieR) checklist and guide. BMJ. 2014:348:g1687.
28. Chan A-W, Tetzlaff JM, Altman DG, Laupacis A, Gøtzsche PC, Krleža-Jerić K, et al. SPIRIT 2013 Statement: Defining Standard Protocol Items for Clinical Trials. Annals of Internal Medicine. 2013;158(3):200-7.
29. Chan A-W, Tetzlaff JM, Gøtzsche PC, Altman DG, Mann H, Berlin JA, et al. SPIRIT 2013 explanation and elaboration: guidance for protocols of clinical trials. BMJ : British Medical Journal. 2013;346:e7586.
30. Bangladesh Interactive Poverty Maps: The World Bank; 2016 [Available from: http://www.worldbank.org/en/data/interactive/2016/11/10/bangladesh-poverty-maps.
31. Poverty Maps of Bangladesh 2010. World Food Programme (WFP); 2010 2010.
32. García-Moreno C, Jansen HAFM, Ellsberg M, Heise L, Watts C. WHO multi-country study on women's health and domestic violence against women. Geneva: World Health Organization; 2005.
33. Report on Violence Against Women Survey 2015. Dhaka, Bangladesh: Bangladesh Bureau of Statistics, Statistics and Informatics Division, Ministry of Planning, Government of the People's Republic of Bangladesh.
34. Reynolds WM. Development of reliable and valid short forms of the marlowe-crowne social desirability scale. Journal of Clinical Psychology. 1982;38(1):119-25.
35. Bergen N, Labonté R. "Everything Is Perfect, and We Have No Problems": Detecting and Limiting Social Desirability Bias in Qualitative Research. Qualitative Health Research. 2020;30(5):783-92.
36. Ellsberg M, Heise L. Researching Violence Against Women: A Practical Guide for Researchers and Activists. Washington DC, United States: World Health Organization, PATH; 2005.
37. Kabeer N. Resources, Agency, Achievements: Reflections on the measurement of Women's Empowerment. Development and Change. 1999;30(3):435-64.
38. Kabeer N. Gender equality and women's empowerment: A critical analysis of the third millennium development goal 1. Gender & Development. 2005;13(1):13-24.
39. Navid RT, Samuels F, Le MAsson V, Talukder A, Gupta T, Yount KM. Understanding intimate partner violence in rural Bangladesh: Prevention and response. London: ODI, Emory University, icddr,b; 2017 May 2017.

For peer review only - http://bmjopen.bmj.com/site/about/guidelines.xhtml

40. Hayes R, Moulton L. Cluster Randomised Trials. New York, USA: Taylor & Francis; 2009.
41. Braun V, Clarke V. Using thematic analysis in psychology. Qualitative Research in Psychology. 2006;3(2):77-101.
42. Guion L, Diehl D, McDonald D. Triangulation: Establishing the Validity of Qualitative Studies. University of Florida, The Institute of Food and Agricultural Sciences (IFAS) 2011.
43. Kim JC, Watts CH, Hargreaves JR, Ndhlovu LX, Phetla G, Morison LA, et al. Understanding the impact of a microfinance-based intervention on women's empowerment and the reduction of intimate partner violence in South Africa. Am J Public Health. 2007;97(10):1794-802.
44. Kabeer N. Women's Empowerment, Development Interventions and the Management of Information Flows. Institute of Development Studies. 2010;41(6).
45. Malhotra A, Schuler SR. Women's Empowerment as a Variable in International Development. In: Bank TW, editor. Measuring Empowerment: Cross Disciplinary Perspectives2005. p. 71- 88.
46. Heymann J, Levy JK, Bose B, Ríos-Salas V, Mekonen Y, Swaminathan H, et al. Improving health with programmatic, legal, and policy approaches to reduce gender inequality and change restrictive gender norms. The Lancet. 2019;393(10190):2522-34.
47. Cunningham K, Ruel MT, Ferguson E, Uauy R. Women's empowerment and child nutritional status in South Asia: a synthesis of the literature. Maternal & Child Nutrition. 2015;11(1):1-19.
48. Yount KM, Cheong YF, Maxwell L, Heckert J, Martinez EM, Seymour G. Measurement Properties of the Project-Level Women's Empowerment in Agriculture Index. International Food Policy Research Institute; 2019.
49. WEAI FAQs: International Food Policy Research Institute 2018 [Available from: https://weai.ifpri.info/weai-resource-center/faqs-2/.
50. Alkire S. Multidimensional Poverty Measures as Relevant Policy Tools. Oxford Poverty & Human Development Initiative 2018.

For peer review only - http://bmjopen.bmj.com/site/about/guidelines.xhtml

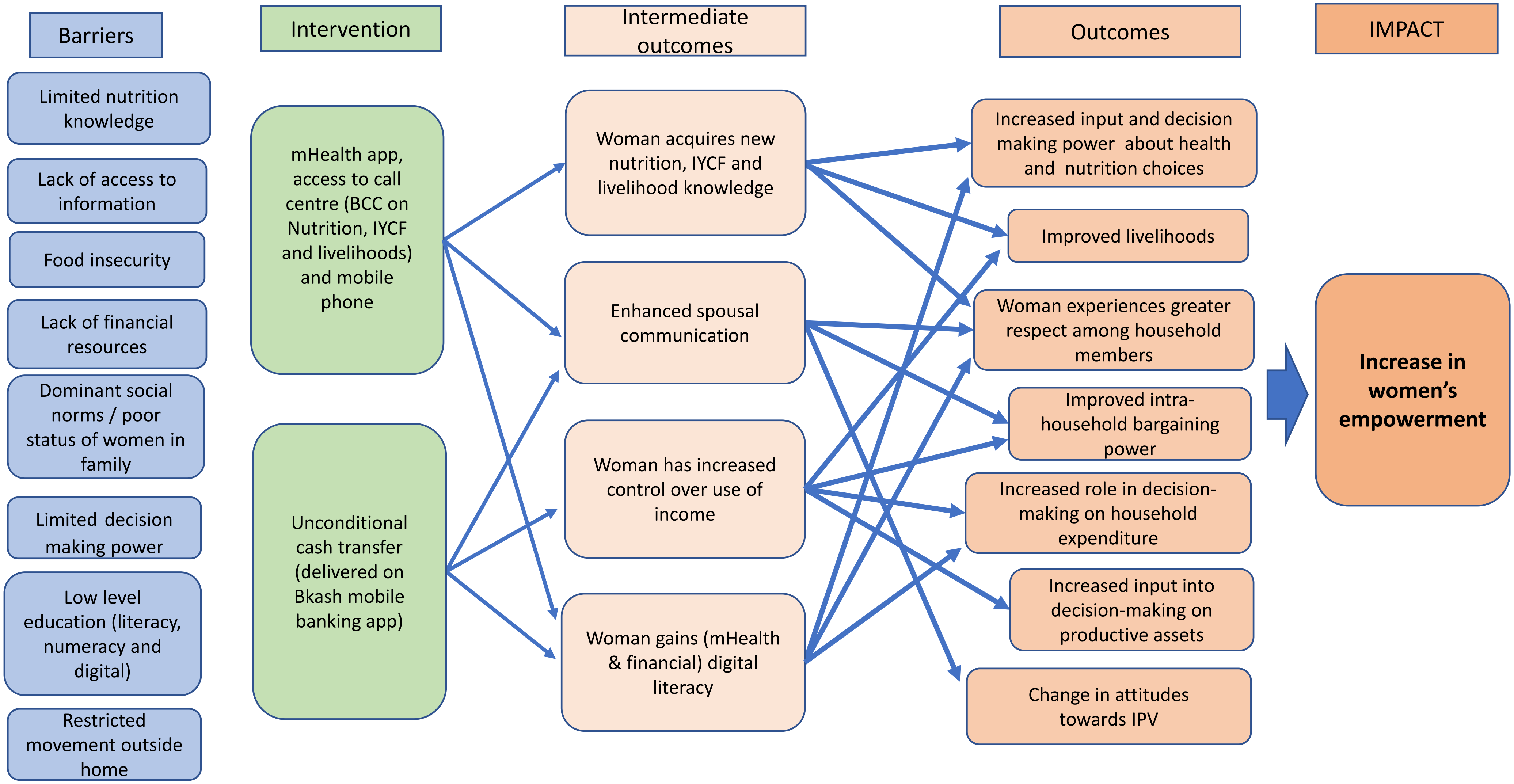
Barriers
Limited nutrition knowledge
Lack of access to information
Food insecurity
Lack of financial resources
Dominant social norms / poor status of women in family
Limited decision making power
Low level education (literacy, numeracy and digital)
Restricted movement outside home
Intervention
mHealth app, access to call centre (BCC on Nutrition, IYCF and livelihoods) and mobile phone
Unconditional cash transfer (delivered on Bkash mobile banking app)
Intermediate outcomes
Woman acquires new nutrition, IYCF and livelihood knowledge
Enhanced spousal communication
Woman has increased control over use of income
Woman gains (mHealth & financial) digital literacy
Outcomes
Increased input and decision making power about health and nutrition choices
Improved livelihoods
Woman experiences greater respect among household members
Improved intra-household bargaining power
Increased role in decision-making on household expenditure
Increased input into decision-making on productive assets
Change in attitudes towards IPV
IMPACT
Increase in women's empowerment

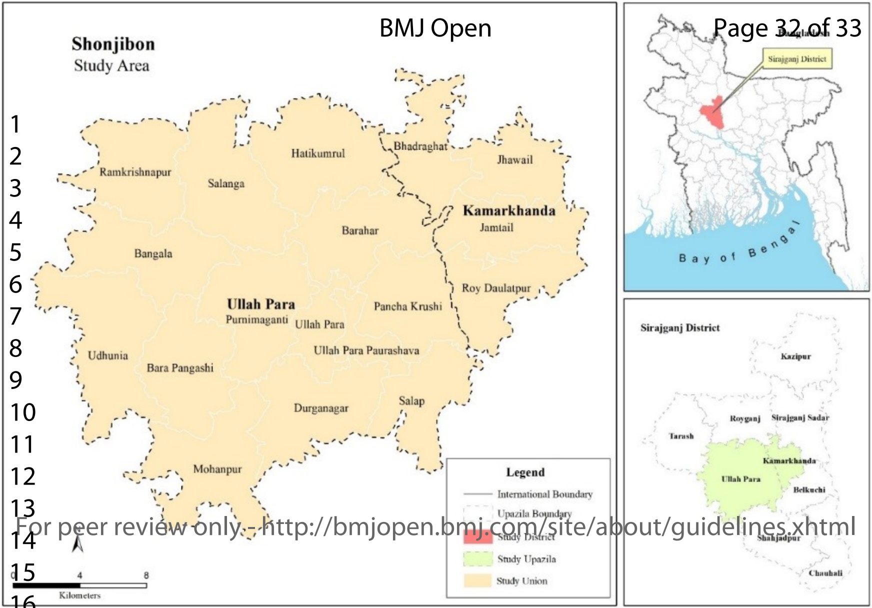

For peer review only - http://bmjopen.bmj.com/site/about/guidelines.xhtml

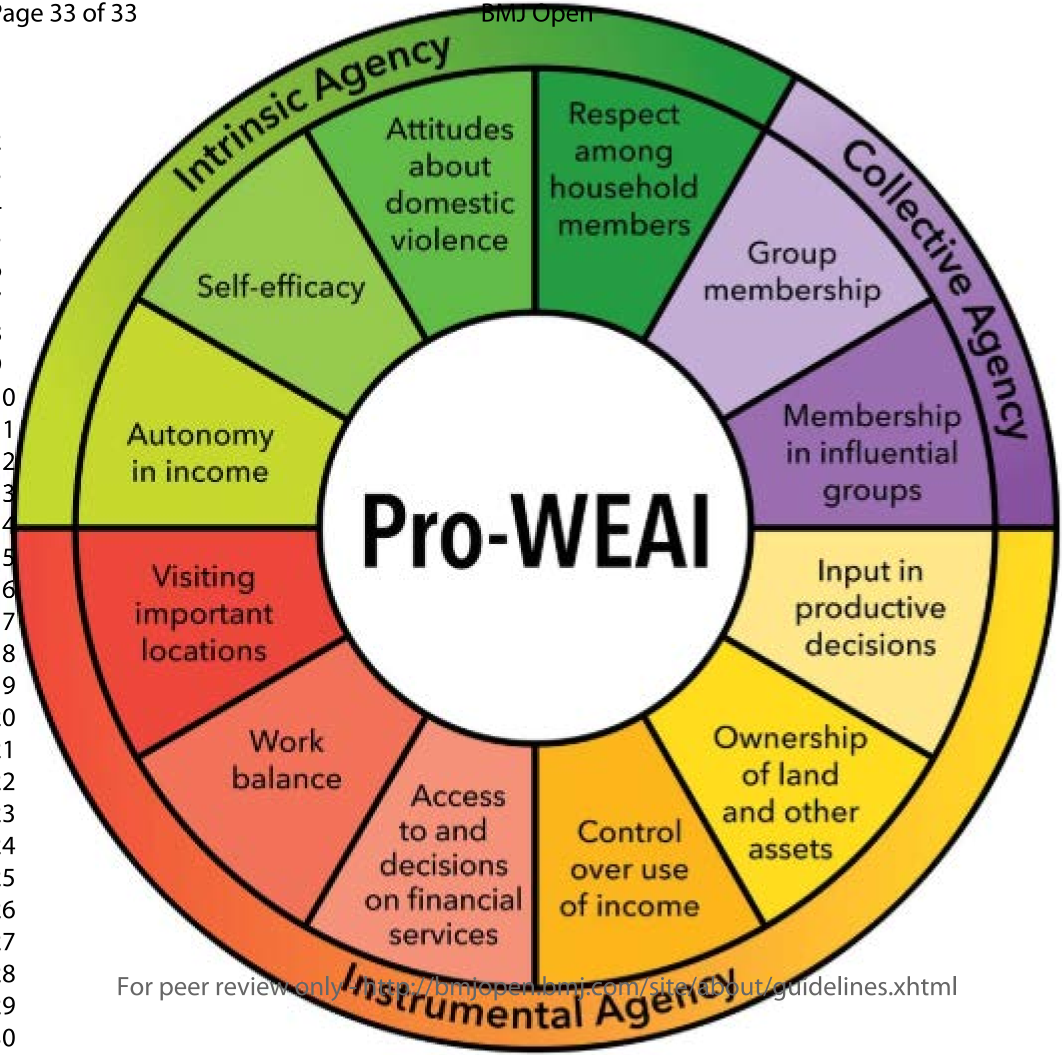
Intrinsic Agency
Collective Agency
Instrumental Agency
Pro-WEAI
Attitudes about domestic violence
Respect among household members
Self-efficacy
Group membership
Autonomy in income
Membership in influential groups
Visiting important locations
Input in productive decisions
Work balance
Ownership of land and other assets
Access to and decisions on financial services
Control over use of income

Appendix

Table 1: SCC women's empowerment summary index components

Shonjibon Trial resources	Pathway	Outcome variable / index components
mHealth app, access to call centre (BCC on Nutrition, IYCF and livelihoods) and mobile phone<br><br>Unconditional cash transfer (delivered on Bkash mobile banking app)	Instrumental Agency (Power to)	1. Control over use of income (G2)<br>2. Input in production decisions (G2)
	Intrinsic agency (power within)	3. Autonomy in income (G8(a))<br>4. Decision-making power on nutrition and health care (X)<br>5. Respect among household members (G7)<br>6. Attitudes toward IPV (G9)

Table 2: Data collection timeline

Methods	Participants	timeline
Semi-structured in-depth interview at the household level		
In-depth interviews with women	1) Pregnant women<br>2) Mothers of children <1 month<br>3) Mothers 1-5 months<br>4) Mothers of children 6 – 59 months	➢ At the start of SCC Trial<br>➢ Endline
In-depth interviews with family members	1) Mother or mother-in-law of women<br>2) Husband of women	➢ At the start of SCC Trial<br>➢ Endline

For peer review only

	3) Other key family members as directed by the woman interviewed	
Pro-WEAI Survey questions		
Survey questionnaires	Female intervention recipients	➢ Baseline<br>➢ Endline

Table 3: Social Desirability Bias

**Directions: Read each item and decide whether it is true (T) or false (F) for you.**			
		**True**	**False**
1	It is sometimes hard for me to go on with my work if I am not encouraged.		
2	I sometimes feel resentful when I don't get my way		
3	On a few occasions, I have given up doing something because I thought too little of my ability		
4	There have been times when I felt like rebelling against people in authority even though I knew they were right		
5	No matter who I'm talking to, I'm always a good listener		
6	There have been occasions when I took advantage of someone		
7	I'm always willing to admit it when I make a mistake		
8	I sometimes try to get even rather than forgive and forget		
9	I am always courteous, even to people who are disagreeable		
10	I have never been irked when people expressed ideas very different from my own		
11	There have been times when I was quite jealous of the good fortune of others		
12	I am sometimes irritated by people who ask favours of me		
13	I have never deliberately said something that hurt someone's feelings		

For peer review only

For peer review only - http://bmjopen.bmj.com/site/about/guidelines.xhtml
